# A general method for calculating power for GEE analysis of complete and incomplete stepped wedge cluster randomized trials

**DOI:** 10.1177/09622802221129861

**Published:** 2022-10-17

**Authors:** Ying Zhang, John S Preisser, Elizabeth L Turner, Paul J Rathouz, Mark Toles, Fan Li

**Affiliations:** 1 Department of Biostatistics, University of North Carolina, Chapel Hill, NC, USA; 2 Department of Biostatistics and Bioinformatics, Duke University, Durham, NC, USA; 3 Department of Population Health, The University of Texas at Austin, Austin, TX, USA; 4 School of Nursing, University of North Carolina, Chapel Hill, NC, USA; 5 Department of Biostatistics, Yale School of Public Health, New Haven, CT, USA; 6 Center for Methods in Implementation and Prevention Science, Yale School of Public Health, New Haven, CT, USA

**Keywords:** Correlated binary outcomes, finite-sample correction, group randomized trial, intraclass correlation, correlation decay

## Abstract

Stepped wedge designs have uni-directional crossovers at randomly assigned time points (steps) where clusters switch from control to intervention condition. Incomplete stepped wedge designs are increasingly used in cluster randomized trials of health care interventions and have periods without data collection due to logistical, resource and patient-centered considerations. The development of sample size formulae for stepped wedge trials has primarily focused on complete designs and continuous responses. Addressing this gap, a general, fast, non-simulation based power procedure is proposed for generalized estimating equations analysis of complete and incomplete stepped wedge designs and its predicted power is compared to simulated power for binary and continuous responses. An extensive set of simulations for six and twelve clusters is based upon the Connect-Home trial with an incomplete stepped wedge design. Results show that empirical test size is well controlled using a t-test with bias-corrected sandwich variance estimator for as few as six clusters. Analytical power agrees well with a simulated power in scenarios with twelve clusters. For six clusters, analytical power is similar to simulated power with estimation using the correctly specified model-based variance estimator. To explore the impact of study design choice on power, the proposed fast GEE power method is applied to the Connect-Home trial design, four alternative incomplete stepped wedge designs and one complete design.

## 1 Introduction

### 1.1 Incomplete stepped-wedge trials

A stepped-wedge cluster randomized trial (SW-CRT) is a type of uni-directional crossover design in which clusters switch from control condition to treatment at randomly assigned time points.^1^ There are three primary types of stepped wedge designs, cross-sectional, closed-cohort and open-cohort designs, which have different schedules of recruiting participants.^2^ Cross-sectional designs recruit a unique set of individuals in each period, whereas closed-cohort designs follow the same individuals in clusters with repeated observations across periods. The open-cohort design, however, allows the attrition of members from and addition of new members to an existing cohort in each period. We will mainly focus on the cross-sectional and closed-cohort designs in this article. Most study planning methods are for complete SW-CRTs where all clusters have outcome data in all periods. However, incomplete stepped wedge designs are increasingly used in cluster randomized trials, whereby some cluster-periods do not record data due to logistical, resource and patient-centered considerations. Specifically, researchers do not collect data in a closed-cohort design or enroll new participants in a cross-sectional design during some cluster-periods. Hemming et al.^3^ described two types of incompleteness in stepped wedge designs, one involving implementation periods and the other staggered study entry or termination of clusters. In the first instance, implementation periods are the transition phases between control and intervention periods in which no data collection or participant enrollment occurs because a block of time is needed before the intervention can be implemented. In the second instance, when the initiation of baseline data collection or study termination is delayed for some clusters according to different periods, there will be staggered entry or end-of-follow-up, correspondingly. Both types of incompleteness may be introduced in the design of a single study. There is a need for the explication of statistical methodology and the development of software tools for a general, non-simulation-based, power calculation procedure based on the generalized estimating equations (GEE) to quickly and accurately calculate power for incomplete stepped wedge designs with a small or large number of clusters.

### 1.2 Population-averaged models for stepped-wedge CRTs

Generalized Linear Mixed Models (GLMMs) based on the maximum likelihood estimation methods and population-averaged (PA) models estimated with GEE are the two most widely used statistical analysis methods for stepped wedge and other CRTs.^4^ Use of GLMMs with non-identity link functions in the analysis of CRTs carries a couple of important caveats. First, the interpretation of the intervention effect is dependent upon the random effects included in the GLMM. For example, the treatment effect parameter in the logistic random effects model for binary outcomes in SW-CRTs has a conditional odds ratio interpretation. Whereas the intervention effect odds ratio is cluster-specific in a random intercept model, its interpretation in a model including cluster-period random effects applies to a conceptual population of cluster-periods possessing the same values of the latent random-effect variables.^5^ Second, while GLMMs are flexible insofar as accounting for the dependence of observations within clusters via random effects, with few exceptions, they do not adequately describe the pattern and magnitude of within-cluster correlation on the natural measurement scale of the responses. This is because exact expressions for the marginal mean and the marginal within-cluster correlation structure for CRTs and SW-CRTs are generally lacking.^6–8^ Perhaps for this reason, while GLMMs are frequently used in the analysis of SW-CRTs with binary and other categorical responses, they are seldom used as the basis for planning SW-CRTs other than computing power by simulation.^9^

Population-averaged models have several advantages for the design and analysis of SW-CRTs. Because SW-CRTs are often used in health care research to inform policy decisions, marginal models carry a straightforward PA interpretation and may be preferred in such settings.^10–12^ In contrast to GLMMs, the intervention effect from a marginal (PA) model describes how the average response changes across the subsets of population defined by the treated and control cluster-periods. Because models for the mean and correlation structures are separately specified, the interpretation of the marginal mean regression parameters remains the same regardless of the correlation specification. The link function is chosen to obtain inference on the target parameters of choice; for binary responses, this could be the odds ratio via the logit link, the risk ratio via the log link, or the risk difference via the identify link. A second advantage in using GEE for SW-CRTs is that inference for mean model parameters is robust to misspecification of the ICC structure in large samples when the sandwich variance estimator is used.^12^ Feng et al.^13^ conducted extensive simulation studies to show that sandwich variance estimator with GEE analysis had good performance for CRTs with 50 or more clusters, even with the misspecification of correlation structures. A third advantage of marginal models is that, unlike GLMMs, they produce estimates of correlations that can be directly used in sample size determination for several outcome types and target parameters for intervention effectiveness, which addresses Item 7a of the CONSORT extension for the SW-CRT^14^ that calls for reporting of “assumptions made about correlations between responses of participants from the same cluster.” For the aforementioned reasons, there is a need for the development of statistical power methods and computational tools based on marginal models for discrete and continuous responses in planning and analysis of SW-CRTs for a selection of intra-cluster correlation structures that reflect the longitudinal and clustered aspects of complete and incomplete cross-sectional and closed-cohort designs.

A distinguishing characteristic of SW-CRTs and other CRTs, which should be considered in statistical analysis, is that they frequently have a small number of clusters.^15^ Barker et al.^4^ found 46/102 had fewer than 10 clusters and Grayling et al.^16^ reported that the median number of clusters in SW-CRTs is 21. For their analysis with marginal models, use of bias-corrected sandwich variance estimators is recommended to improve the accuracy of inference regarding tests of the intervention effect including control of Type I error.^10,17^ In simulation studies, these bias-corrected GEE methods performed similarly well under correctly specified and mis-specified ICC matrices for as few as 10 clusters.^12,18^ Additionally, to address the finite-sample bias of correlation parameter estimates, Matrix-adjusted Estimating Equations (MAEEs) incorporates a matrix-based finite-sample bias adjustment in a second set of estimating equations for correlation parameters solved simultaneously with the usual GEE for the marginal mean parameters.^10,19^

### 1.3 Sample size and power methods for SW-CRTs

Because CRTs are less powerful than individually randomized trials, determination of the proper number and allocation of study participants in SW-CRTs is critically important. However, most sample size methods for SW-CRTs are for continuous responses and complete designs. In particular, there is substantial literature on sample size and power calculation procedures for continuous responses in complete SW-CRTs based on linear mixed models (LMMs) estimated by maximum likelihood or generalized least squares.^1,5,20–22^ In the random-cluster-intercepts linear model^1^ and a nested exchangeable three-level model extension,^23^ Hemming et al.^3^ developed formulae for statistical power for continuous responses through z-statistics for single degree-of-freedom contrasts for the intervention effect in the SW-CRTs, which they adapt to incomplete designs. Furthermore, Hemming et al.^24^ implemented the power and sample size calculation procedures for CRTs with linear mixed models into an R shiny app for complete and incomplete SW-CRT.

In the case of population-averaged models, simple-to-use sample size formulae based on PA models for continuous responses and non-simulation procedures for binary responses have recently been proposed for complete, cross-sectional and closed-cohort SW-CRTs within the framework of GEE.^10^ The methods extend earlier sample size formulae for GEE analysis of parallel-groups CRTs, including cross-sectional and cohort CRTs^25,26^ and multi-level CRTs.^27,28^ Prior work^10,29^ has shown that the analytical power for marginal mean (e.g. intervention) parameters in complete SW-CRTs agrees well with simulated power for as few as eight clusters, when PA models are analyzed using GEE with bias-corrected sandwich standard errors concurrently with MAEE estimation of ICCs.^19^ Chen et al.^30^ proposed a R package swdpwr to calculate the analytical power for complete SW-CRTs with binary or continuous outcomes.

In this article, computationally fast, non-simulation procedures for determining sample size and statistical power for GEE analysis of binary and continuous responses are extended from complete SW-CRTs in Li et al.^10^ to SW-CRTs with incomplete designs. The article is organized as follows. Section 2, motivates our work with the description of a real-world small sample incomplete stepped wedge design. Section 3, describes the population-averaged models of interest consisting of marginal mean and within-cluster correlation models with special consideration of correlation structures suitable for cross-sectional and closed-cohort SW-CRTs, respectively. Estimation is presented within a GEE framework involving a pair of estimating equations for the marginal means and correlation parameters. To accommodate a small number of clusters, MAEE for correlation parameter estimation and bias-corrected covariance estimators are employed. Section 4, proposes the general power procedure for GEE analysis of discrete and continuous responses for completed and incomplete stepped wedge designs. Section 5, presents a simulation study of empirical type I error and power based on GEE/MAEE analysis for incomplete SW-CRTs based on Connect-Home trial with six or 12 clusters, which is compared to predicted power from the non-simulation fast GEE power method.^10^ Section 6, describes the application of the proposed fast GEE method to study planning based on the Connect-Home trial and the final section discusses results and their implications.

## 2 Motivating example: The Connect-Home trial

**Figure 1.**
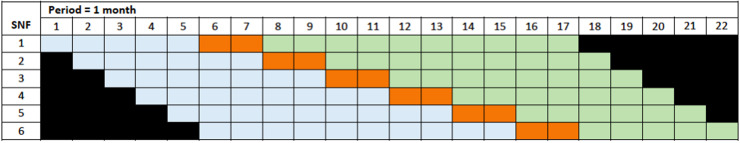
The study design of the Connect Home trial: the blue, orange and green cells denote control, implementation and intervention cluster-periods, respectively.

The Connect-Home trial uses an incomplete, cross-sectional stepped wedge design to test an intervention to improve outcomes for rehabilitation patients transitioning from skilled nursing facilities (SNFs) to home-based care.^31^ The primary component of the intervention is an individualized Transition Plan of Care that SNF staff create to support the patient and caregiver at home. The incomplete design with six SNFs (clusters) and four patients per cluster-period (360 patients total), shown in Figure 1, was chosen based on considerations of internal validity and power under restrictions placed by available resources and logistical considerations. The black and orange boxes represent cluster-periods where no patients are enrolled, giving an incomplete design. Staggered enrollment of SNFs (clusters) is used to initiate data collection in stages with limited research staff resulting in the black boxes. The rationale for the orange boxes representing the implementation phase is that two months are needed to activate the intensive intervention through training nursing home and home health care staff. The analysis for Connect-Home specifies linear mixed models to compare observations between intervention and usual care periods for the study’s two primary responses, which are continuous measures of patient and caregiver preparedness for discharge collected at seven days post-discharge. Considering that marginal models^25^ are particularly useful for discrete responses when population-averaged interpretations are desired, this article focuses on binary responses, although continuous responses are also considered.

Our investigation considers that the Connect-Home study design is distinct from that of the typical SW-CRT in several aspects. First, the number of periods (J=22) is much greater than the number of steps (S=6), as compared to the common restriction of J=S+1.^1,3,10^ Next, the number of SNFs or clusters (I=6) in the Connect-Home SW-CRT is much fewer than the number periods, which not only has implications for the specification of the time trend in the model, necessary for a valid analysis, but also invites detailed examination of the finite-sample performance of treatment effect estimators. Notwithstanding the particularities of the Connect-Home stepped wedge trial, the general power calculation methodology described in the next section has very broad application to complete and incomplete longitudinal cluster randomized trial designs.

## 3 GEE analysis of stepped-wedge designs

### 3.1 Marginal model approach

A unifying population-averaged model framework is described for the design and statistical analysis of stepped wedge designs. The following notations apply to both cross-sectional and cohort SW-CRT designs where there are J periods, S sequences, I clusters and $m_{s}$ clusters in sequence s, such that $I=\sum_{s=1}^{S}m_{s}$. Let $y_{ijk}$ denote the response of the individual k from cluster i during timepoint j for $i=1\ldots I,j=1\ldots J_{i}$, and $k=1\ldots N_{ij}$, noting that $J_{i}\leq J$ is the number of observed periods (i.e. with data collection) for cluster i and $N_{ij}$ is the cluster-period size. Let $\mu_{ijk}$ denote the marginal mean response of $y_{ijk}$, which is related to the intervention and calendar period via the model

(1) $$g(\mu_{ijk})=\beta_{0}+\beta_{1}(t_{ij}-1)+u_{ij}\delta$$

where g is a link function, $\beta_{0}$ is the intercept, the $\left\{t_{ij}\;:\;j=1,\ldots ,J_{i}\right\}$ are integer-valued calendar periods from the study design such that $\beta_{1}$ is the increment in the mean response on the scale of the link function for a unit increase in calendar period, and $u_{ij}$ is the treatment status in cluster i at time point j. For example, a cluster from the second sequence in Figure 1 would have timepoints indexed by {j:1,…,6,7,…,15} corresponding to calendar periods $\left\{t_{ij}\;:\;2,\ldots ,7,10,\ldots ,18\right\}$. To define a class of designs for which Figure 1 is an archetype allowing for implementation periods and/or staggered entry/termination, let $b_{i0}$ and $b_{i1}$ denote the first and last calendar periods of data collection for cluster i in the control condition ( $b_{20}=2$ and $b_{21}=7$, continuing the example of the second sequence, s=2) such that there are $b_{i}=b_{i1}-b_{i0}+1$ total periods in the control condition; $q_{i0}$ and $q_{i1}$ as the first and last calendar periods of data collection for cluster i in the intervention condition (e.g. $q_{20}=10$ and $q_{21}=18$, s=2) such that there are $q_{i}=q_{i1}-q_{i0}+1$ total periods in the intervention condition; and $c_{i}$ implementation periods occurring in calendar periods $b_{i1}+1,\ldots b_{i1}+c_{i}$ where $c_{i}=q_{i0}-b_{i1}-1$; all of the study designs investigated in this article have a constant $c_{i}=c$ across clusters. In Figure 1, c=2 with values $\left\{(b_{i0},b_{i1},q_{i0},q_{i1})\;:\;i=1,\ldots ,I\right\}$ given in the Web Appendix A. Note that for open cohort designs, we could write $t_{ijk}$ in equation (1) instead of $t_{ij}$ to allow individuals within the same cluster to have different sets of time points.^32^ However, we mainly focus on cross-sectional and closed-cohort designs in this article and assume a common set of time points.

Two types of marginal mean models are investigated in the article, the widely used the *average intervention effects model*^1,3,10^ and the *incremental intervention effects model*.^33^ In the average intervention effects model, $u_{ij}$ is the period-specific treatment indicator (1 = intervention; 0 = control) for cluster i and δ is the intervention effect, irrespective of time on treatment, on the link function scale. Conversely, the incremental intervention effects model assumes a gradual uptake of the intervention such that its effect depends on time-on-treatment. In this model, $u_{ij}=0$ for control periods $j=1,\ldots ,b_{i}$, whereas, for intervention periods, $u_{ij}=(j-b_{i})/q,j=b_{i}+1,\ldots ,J_{i}$ where $J_{i}=b_{i}+q_{i}$ and q>0, whose value is chosen to scale the intervention effect δ according to user preference. In this article, q=10 so that δ is defined as the intervention effect on the link function scale after 10 periods, which corresponds to the number of intervention periods for the first SNF in the Connect-Home trial (Figure 1). Finally, we note that equation (1) specifies linear period effects, which is specifically motivated by trial designs like Figure 1 where J>I. Specification of linear period effects gives positive cluster level degrees of freedom I−3 when I>3 for testing the intervention effect via $H_{0}$: δ=0 as described in section 4. On the other hand, model equation (1) with categorical period effects would have I−(J+1) degrees of freedom, which is positive only if I>J+1.^10^ While not considered in this article, other models are possible such as those with higher order polynomials (e.g. quadratic, cubic terms) for time.

Specification of the marginal model is completed with the covariance structure of a cluster’s response. The variance of the response is $\text{var}(y_{ijk})=v_{ijk}\phi$ where $v_{ijk}$ is the variance function and ϕ is the dispersion parameter. For binary responses, $v_{ijk}=\mu_{ijk}(1-\mu_{ijk})$ and ϕ=1, while for continuous responses following typical normal model assumptions, $v_{ijk}=1$ and ϕ is the constant variance. In this article, five within-cluster correlation structures commonly used or recommended for SW-CRTs are considered (Table 1). Specifically, there are two distinct correlation structures for each of the closed-cohort and cross-sectional design, and the unrealistic (but frequently used) exchangeable structure for the both designs. Each structure incorporates the usual ICC, which measures the correlation between responses from different individuals within the same cluster during the same period: $\mathrm{corr}(y_{ijk},y_{ijk^{\prime }})=\alpha_{0}$, $k\neq k^{\prime }$. For cross-sectional designs, the nested exchangeable correlation structure additionally specifies a correlation parameter $\alpha_{1}$ for observation pairs collected from different periods. Alternatively, exponential decay assumes the between-period correlation between responses from different individuals within the same cluster in the jth and $j^{\prime }$th periods decays over time as $\alpha_{0}\rho^{\left|t_{ij}-t_{ij^{\prime }}\right|}$. For closed-cohort designs, the block exchangeable correlation structure distinguishes between-period correlations for pairs of individuals, $\alpha_{1},$ from a constant intra-individual correlation for repeated observations, $\alpha_{2}$.^19,10^ On the other hand, the proportional decay correlation structure^29,34^ allows for correlation decay over time, where the intra-individual correlation $\mathrm{corr}(y_{ijk},y_{ij^{\prime }k})=\rho^{\left|t_{ij}-t_{ij^{\prime }}\right|},j\neq j^{\prime }$ has a first-order auto-regressive structure decay rate ρ and the between-period correlation among responses from different individuals within the same cluster is $\alpha_{0}\rho^{\left|t_{ij}-t_{ij^{\prime }}\right|},j\neq j^{\prime },k\neq k^{\prime }$. Note that the nested exchangeable correlation for cross-sectional designs is a special case of block exchangeable correlation when $\alpha_{1}=\alpha_{2}$. Finally, the exchangeable correlation^1^ specifies that within- and between-period correlations are equal. For cross-sectional designs, this means that $\alpha_{0}=\alpha_{1}$ and for cohort designs, $\alpha_{0}=\alpha_{1}=\alpha_{2}$. Examples of correlation matrices are listed in Web Appendix B.

**Table 1.** Intra-cluster correlation structures in marginal models for stepped-wedge cluster randomized trials (SW-CRTs) (*i* = cluster, *j* = period, *k* = individual).

Design	Correlation structure	Model
Special case ${}^{a}$	Exchangeable	$\mathrm{corr}\left(y_{ijk},y_{ijk^{\prime }}\right)=\alpha_{0}$
		$\mathrm{corr}\left(y_{ijk},y_{ij^{\prime }k^{\prime }}\right)=\alpha_{0}$
Cross sectional	Nested exchangeable	$\mathrm{corr}\left(y_{ijk},y_{ijk^{\prime }}\right)=\alpha_{0}$
		$\mathrm{corr}\left(y_{ijk},y_{ij^{\prime }k^{\prime }}\right)=\alpha_{1}$
	Exponential decay	$\mathrm{corr}\left(y_{ijk},y_{ijk^{\prime }}\right)=\alpha_{0}$
		$\mathrm{corr}(y_{ijk},y_{ij^{\prime }k^{\prime }})=\alpha_{0}\rho^{\left|t_{ij}-t_{ij^{\prime }}\right|}$
Cohort	Block exchangeable	$\mathrm{corr}\left(y_{ijk},y_{ijk^{\prime }}\right)=\alpha_{0}$
		$\mathrm{corr}\left(y_{ijk},y_{ij^{\prime }k^{\prime }}\right)=\alpha_{1}$
		$\mathrm{corr}\left(y_{ijk},y_{ij^{\prime }k}\right)=\alpha_{2}$
	Proportional decay	$\mathrm{corr}(y_{ijk},y_{ijk^{\prime }})=\alpha_{0}$
		$\mathrm{corr}(y_{ijk},y_{ij^{\prime }k^{\prime }})=\alpha_{0}\rho^{\left|t_{ij}-t_{ij^{\prime }}\right|}$
		$\mathrm{corr}(y_{ijk},y_{ij^{\prime }k})=\rho^{\left|t_{ij}-t_{ij^{\prime }}\right|}$

${}^{a}$Exchangeable correlation is a special case of cross-sectional and cohort designs that assumes no correlation decay.

The GEE/MAEE method formalizes estimation of correlation parameters through specification of a second generalized linear model for intracluster correlations:

$$h(\rho_{ijkj^{\prime }k^{\prime }})=z_{ijkj^{\prime }k^{\prime }}^{\prime }\;\alpha$$

where h(.) is the link function, α is the correlation parameter vector, and $z_{ijkj^{\prime }k^{\prime }}$ is a covariate vector for the pairwise correlation $\rho_{ijkj^{\prime }k^{\prime }}$ of the observation pair denoted by double indices (j,k), and $\left(j^{\prime },k^{\prime }\right)$. While various link functions can be applied, under block exchangeable correlation, the identity link function,^10,19^ $h(\rho_{ijkj^{\prime }k^{\prime }})=\rho_{ijkj^{\prime }k^{\prime }},$ gives the model:

(2) $$\rho_{ijkj^{\prime }k^{\prime }}=z_{0ijkj^{\prime }k^{\prime }}\alpha_{0}+z_{1ijkj^{\prime }k^{\prime }}\alpha_{1}+z_{2ijkj^{\prime }k^{\prime }}\alpha_{2}$$

with $z_{0ijkj^{\prime }k^{\prime }}=1$ for two observations coming from the same period $j=j^{\prime }$ and 0 otherwise; $z_{1ijkj^{\prime }k^{\prime }}=1$ for two observations coming from the different period and different individuals $j\neq j^{\prime }\mathrm{and}\;k\neq k^{\prime }$, 0 otherwise; $z_{2ijkj^{\prime }k^{\prime }}=1$ for two observations coming from the same individual $j\neq j^{\prime }\mathrm{and}\;k=k^{\prime }$, 0 otherwise. The block exchangeable reduces to the nested and exchangeable correlation structures as noted above. Owing to the exponential form of the correlation decay structures, a log link, $h(\rho_{ijkj^{\prime }k^{\prime }})=\log\;(\rho_{ijkj^{\prime }k^{\prime }}),$ is used such that $\log\;(\rho_{ijkj^{\prime }k^{\prime }})=z_{0ijkj^{\prime }k^{\prime }}\gamma_{0}+z_{1ijkj^{\prime }k^{\prime }}\gamma_{1}$ where $z_{0ijkj^{\prime }k^{\prime }}=1$ if $k\neq k^{\prime }$ (and 0 otherwise) is an indicator for two observations coming from different patients and $z_{1ijkj^{\prime }k^{\prime }}=|t_{ij}-t_{ij^{\prime }}|$ is the absolute distance between two observations with respect to calendar periods of measurement. Because $\alpha_{0}=e^{\gamma_{0}}$ and $\rho =e^{\gamma_{1}},$ MAEE, described in the next section, can be used to estimate $\gamma_{0}$ and $\gamma_{1}$ in the exponential and proportional decay ICC structures. Moreover, confidence intervals can be obtained for them by exponentiating the confidence limits for $\gamma_{0}$ and $\gamma_{1}$, respectively, or through application of the delta method to obtain their bias-corrected standard errors.

### 3.2 Estimation procedure with finite-sample bias corrections

Estimation of marginal models for SW-CRTs is based on GEE with MAEE^19^ to reduce the finite-sample bias of the correlation parameter estimates.^10,29^ We denote the response vector as $y_{i}=(y_{i11},\ldots ,y_{iJ_{i}N_{iJ_{i}}}{)}^{\prime }$ and its expected mean vector as $\mu_{i}=(\mu_{i11},\ldots ,\mu_{iJ_{i}N_{iJ_{i}}}{)}^{\prime }$. The marginal mean model for cluster i can be expressed in vector form: $g(\mu_{i})=X_{i}\theta$, where $X_{i}=(x_{i11},\ldots ,x_{iJ_{i}N_{iJ_{i}}}{)}^{\prime }$ is its $n_{i}\times 3$ covariate matrix and $\theta =(\beta_{0},\beta_{1},\delta )$ is the marginal mean regression parameters. Let $D_{i}=\partial \mu_{i}/\partial \theta^{\prime }$ and $V_{i}=A_{i}^{1/2}R_{i}A_{i}^{1/2}$, in which $R_{i}$ is the working correlation with correlation parameters α and $A_{i}=\phi \mathrm{diag}\{v(\mu_{i11}),\ldots ,v(\mu_{iJ_{i}N_{iJ_{i}}})\}$. Under the marginal model, standard first-order GEE are solved for marginal mean regression parameters θ: $U=\sum_{i=1}^{I}D_{i}^{\prime }V_{i}^{-1}(y_{i}-\mu_{i})=0$. To improve the estimation of ICC parameters, a separate set of estimating equations involving a matrix-based small-sample bias adjustment (MAEE) are solved for correlation parameters. The exact details for the fitting algorithm of GEE/MAEE have been fully presented in Preisser et al.,^19^ which is described briefly in the paper to reduce redundancy. Finally, in the case of continuous responses, an additional step is required to update the dispersion parameter ϕ via the method of moments.^10^

With regard to the variance estimators of the marginal regression parameters in the SW-CRTs, the model-based variance $\Sigma_{1}^{-1}=[\sum_{i=1}^{I}D_{i}^{\prime }V_{i}^{-1}D_{i}{]}^{-1}$ and sandwich variance $\Sigma_{1}^{-1}\Sigma_{0}\Sigma_{1}^{-1}$ are the most commonly used, where

(3) $${\hat{\Sigma }}_{0}=\sum_{i=1}^{I}F_{i}D_{i}^{\prime }V_{i}^{-1}B_{i}\left(y_{i}-{\hat{\mu }}_{i}\right){\left(y_{i}-{\hat{\mu }}_{i}\right)}^{\prime }B_{i}^{\prime }V_{i}^{-1}D_{i}F_{i}$$

with ${\hat{\Sigma }}_{0}$ and ${\hat{\Sigma }}_{1}$ evaluated at the solution of the GEEs ( $\hat{\theta },\hat{\alpha }$). The model-based variance estimator is suitable to estimate the variance of $\hat{\theta }$ when the working correlation is believed to be equal to the true correlation structure. On the other hand, sandwich variance estimators have the virtue that they provide consistent estimates of the variance matrix for parameter estimates even when the assumed variance structure fails to hold.^35^ When both $F_{i}$ and $B_{i}$ are identity matrices, equation (3) reduces to the uncorrected sandwich estimator of Liang and Zeger^36^ referred to as BC0, which tends to underestimate the variances. We also consider three bias-corrected sandwich covariance estimators^10,29^ to reduce finite sample bias. Setting $F_{i}=I$ and $B_{i}=(I-H_{i}{)}^{-1/2}$ gives the finite-sample correction of Kauermann and Carroll^35^ or BC1. in which $H_{i}=D_{i}(\sum_{i=1}^{I}D_{i}^{\prime }V_{i}^{-1}D_{i}{)}^{-1}D_{i}^{\prime }V_{i}-1$ is the leverage matrix defined in Preisser and Qaqish.^37^ Next, setting $F_{i}=I$ and $B_{i}={\left(I-H_{i}\right)}^{-1}$ gives the finite-sample correction of Mancl and DeRouen^38^ or referred as BC2. Because the matrix elements of the cluster leverage are between 0 and 1, there is an order of bias-correction BC0<BC1<BC2 Preisser et al.^19^ Finally, setting $F_{i}=\text{diag}\left\{{\left(1-\min\left\{\zeta ,{\left[D_{i}^{\prime }V_{i}^{-1}D_{i}\Sigma_{1}^{-1}\right]}_{\;jj}\right\}\right)}^{-1/2}\right\}$ and $B_{i}=I$ gives the finite-sample correction of Fay and Graubrad^39^ or BC3, where the bound parameter ζ is a user-defined constant (<1) with a default value 0.75. Analogous finite-sample bias-corrected variance estimators are available for the MAEE correlation parameter estimates;^19^ while finite-sample bias corrections are recommended in GEE/MAEE marginal model analysis for CRTs, they are not required for estimating the sample size and power in the design stage, as discussed in the next section.

## 4 Adaptation of fast GEE power to incomplete designs

### 4.1 Overview of the fast GEE power method for stepped-wedge designs

The z-test of the intervention effect $H_{0}$: δ=0 vs $H_{1}$: δ≠0 is based upon the asymptotic normal distribution of $\sqrt{I}(\hat{\delta }-\delta )$ with mean zero and variance determined by the (3,3)-th element of cov $\left(\sqrt{I}(\hat{\theta }-\theta )\right)$ , when I is sufficiently large, such as I≥18 for SW-CRTs.^10^ In turn, the Wald-test statistic $\hat{\delta }/\mathrm{var}(\hat{\delta })$ has an asymptotically standard normal distribution under the null hypothesis. Thus, power to detect an intervention effect of size δ≠0 with a nominal type I error rate α is $\Phi \left(z_{\alpha /2}+|\delta |/\sqrt{\mathrm{var}(\hat{\delta })}\right)$. However, for CRTs with a small number of clusters, the t-test is a good alternative with power modified as $\Phi_{t,I-p}\left(t_{\alpha /2,I-p}+|\delta |/\sqrt{\mathrm{var}(\hat{\delta })}\right)$. The degrees of freedom of the t-statistic in CRTs is typically set to I−p, where p=dim(θ) is the number of estimated marginal mean model parameters in equation (1), which is p=3. The variance of the intervention effect $\text{var}(\hat{\delta })$ in the determination of power is defined as the (3,3)-th element in the model-based covariance matrix ${\mathrm{cov}}_{\mathrm{MB}}(\hat{\theta })=\Sigma_{1}^{-1}$. We refer to this analytical power method^40^ as fast GEE power or predicted power.

### 4.2 Fast GEE power calculations for incomplete SW-CRTs

The fast GEE power procedure for SW-CRTs has been previously investigated for complete SW-CRTs for the average intervention effects marginal mean model with categorical period effects.^10^ Motivated by the Connect-Home trial, we describe its implementation for incomplete SW-CRTs with application to a marginal mean model with linear period effects. Because the proposed method is based on the general GEE power method of Rochon^40^, it applies to generalized linear models for correlated binary and continuous responses with arbitrary link functions, variance functions and correlation matrices. A key step in the application of the fast GEE power computation is the generation of the cluster-level design matrices, $X_{i},i=1,\ldots ,I$. For cross-sectional and closed-cohort SW-CRTs under model equation (1), we specify a Design Pattern (DP) matrix to represent the experimental design as was done for the power analysis of continuous responses in linear mixed models.^3^ In order to efficiently compute power for incomplete SW-CRT designs, we additionally propose a Completeness Matrix (CM) to represent the planned missingness pattern. Both DP and CM matrices have dimension S x T where each matrix element corresponds to a representative cluster-period in the SW-CRT design. The DP matrix has entries of 0 for control condition, 1 for intervention condition and “.” for cluster-periods in sequences without data collection. The CM matrix is determined from DP and has entries of 0 for cluster-periods without data collection and entries of 1 for those with data collection. To illustrate, the incomplete SW-CRT example of Hemming et al.^3^ has S=2 treatment sequences, T=4 periods, and an implementation period that occurs in period 2 for the first sequence and in period 3 for the second sequence. Using the taxonomy defined in Section 3.1. for incomplete designs, this design is specified by $\left(b_{10},b_{11},q_{10},q_{11})=(1,1,3,4\right)$ for s=1 and $\left(b_{20},b_{21},q_{20},q_{21})=(1,2,4,4\right)$ for s=2. Then

$$\mathrm{DP}=\left(\begin{array}{cccc} 0 & . & 1 & 1\\ 0 & 0 & . & 1 \end{array}\right),\;\mathrm{CM}=\left(\begin{array}{cccc} 1 & 0 & 1 & 1\\ 1 & 1 & 0 & 1 \end{array}\right)$$

The sth row of CM is used to define the incidence matrix $K_{s},s=1,\ldots ,S$, which is applied to the design matrix $X_{s}^{\left(c\right)}$ for each cluster in the sth treatment sequence from a reference complete SW-CRT design, in order to generate the design matrices for the incomplete design of interest with the formula $X_{s}^{\left(inc\right)}=K_{s}X_{s}^{\left(c\right)}$. Thus, the general technique of Rochon^40^ is applied to account for staggered entry/dropout or, as in this example, implementation periods. For an incremental intervention effects model for the SW-CRT design with S=2 sequences and T=4 periods, the design matrices from the reference complete design are:

$$X_{1}^{\left(c\right)}=\left(\begin{array}{ccc} 1 & 0 & 0\\ 1 & 1 & 0\\ 1 & 2 & 1/2\\ 1 & 3 & 1 \end{array}\right),\;X_{2}^{\left(c\right)}=\left(\begin{array}{ccc} 1 & 0 & 0\\ 1 & 1 & 0\\ 1 & 2 & 0\\ 1 & 3 & 1/2 \end{array}\right)$$

where columns correspond to intercept, linear period effects, and incremental intervention effect, respectively; these give the design matrix for a complete design with 0’s replacing “.” in matrix DP defined above. The incidence matrices with indices corresponding to the rows of CM above are:

$$K_{1}=\left(\begin{array}{cccc} 1 & 0 & 0 & 0\\ 0 & 0 & 1 & 0\\ 0 & 0 & 0 & 1 \end{array}\right),\;K_{2}=\left(\begin{array}{cccc} 1 & 0 & 0 & 0\\ 0 & 1 & 0 & 0\\ 0 & 0 & 0 & 1 \end{array}\right)$$

Then, the incomplete design matrices, having three rows corresponding to the three periods of data collection, for clusters in the first sequence and the second sequences, respectively, are

$$X_{1}^{\left(inc\right)}=K_{1}X_{1}^{\left(c\right)}=\left(\begin{array}{ccc} 1 & 0 & 0\\ 1 & 2 & 1/2\\ 1 & 3 & 1 \end{array}\right),\;X_{2}^{\left(inc\right)}=K_{2}X_{2}^{\left(c\right)}=\left(\begin{array}{ccc} 1 & 0 & 0\\ 1 & 1 & 0\\ 1 & 3 & 1/2 \end{array}\right)$$

These two design matrices for individual-level data are replicated according to the number of clusters per treatment sequence and the number of observations per cluster. In a cross-sectional or a closed-cohort design having N individuals per cluster-period, clusters in the first sequence will have design matrices $X_{1}=X_{1}^{\left(inc\right)}⊗1_{N}$ and those in the second sequence will have $X_{2}=X_{2}^{\left(inc\right)}⊗1_{N}$. Finally, the correlation matrix, variance matrix and first-derivative matrix can be generated from the incidence matrix for each cluster by $R_{i}^{\left(inc\right)}=K_{i}R_{i}^{\left(c\right)}K_{i}^{\prime }$, $A_{i}^{\left(inc\right)}=K_{i}A_{i}^{\left(c\right)}K_{i}^{\prime }$, and $D_{i}^{\left(inc\right)}=K_{i}D_{i}^{\left(c\right)}K_{i}^{\prime }$, where $R_{i}^{\left(c\right)},A_{i}^{\left(c\right)},D_{i}^{\left(c\right)}$ are the corresponding matrices of the reference complete SW-CRTs. Thus, the CM matrix determines incidence matrices for a specified marginal model that gives the model-based variance estimator needed to compute power. A SAS macro **CRTFASTGEEPWR**^41^ implements the fast GEE power method for CRTs with complete and incomplete designs that can be described by DP and CM matrices. It is available at http://www.bios.unc.edu/~preisser/personal/software.html.

## 5 Simulation study

Simulation studies are conducted to demonstrate that the proposed fast GEE power method gives predicted power similar to that of time-intensive simulations for binary and continuous responses in incomplete cross-sectional and closed-cohort SW-CRTs. We also compare power between the five correlation structures in Table 1, the two types of marginal mean models in section 3.1. and two intervention effect sizes δ as detailed below. Following Li et al.,^10^ empirical test size and power of GEE Wald-tests are simulated for binary responses and continuous responses. Specifically, motivated by the Connect-Home trial design, data are simulated for sample sizes of 6 or 12 clusters assuming equal allocation of clusters across sequences, with 4 or 2 patients per cluster-period, respectively, so that the total number of patients is fixed at 360. In Web Appendix A, the DP and CM matrices are displayed for the Connect-Home trial. The design matrices $X_{i},i=1,\ldots ,I$ are derived from the DP and CM matrices following the procedure in Section 4.2.

Clustered binary data are randomly generated from marginal models from Section 3.1. with a logit link function for the marginal mean in equation (1) and correlation matrix R(α) by the method of Qaqish.^42^ Baseline prevalence $\exp\;(\beta_{0})/[1+\exp\;(\beta_{0})]$ for all models is chosen as 0.7. We assume a gently decreasing linear period effects such that $\beta_{0}=$ 0.85 and $\beta_{1}=-0.01$ for $t_{ij}=1,\ldots ,22$, calculated from assumptions that baseline prevalence is 0.7 and a negative time trend such that prevalence at the last period (22 months) is 0.65. The effect size in odds ratio exp(δ) is fixed at 1 for studying empirical test size. For studying power under the average intervention effects model, the effect sizes are δ=log(0.3)=−1.2 and δ=log(0.25)=−1.4. Effect sizes at 10 months on treatment (q=10) for the incremental intervention effects model are δ=−1.8 and δ=−2.4. For continuous responses, clustered data will be generated from multivariate normal distributions with an identity link model and variance R(α) where the total variance ϕ = 1. A gently decreasing linear period effects is assumed such that $\beta_{0}=2$ and $\beta_{1}=-0.05$. Under this model, conclusions are expected to be insensitive to the choice of the period effects as long as these effects are accounted for in the GEE analyses, according to the analytical variance formula derived by Li et al.^10^ The effect sizes for the average intervention effects model are δ=0.5 and δ=0.7. Effect sizes for the incremental intervention effects model with q=10 are δ = 0.8 and δ = 1.

In simulation scenarios for both binary and continuous responses, five marginal correlation structures (Table 1) and one set of α values per structure are chosen. For the nested exchangeable case, α=(0.03,0.015); for exchangeable correlation $\alpha_{0}=0.03$; for the exponential decay, $\alpha_{0}=0.03$ and ρ=0.8, which represents moderate correlation decay over time; for the block exchangeable case, α=(0.03,0.015,0.2) as in Li et al.^10^; for the proportional decay case, $\alpha_{0}=0.03$, ρ=0.7. For each scenario, 1000 data replicates are generated to fit GEE for the marginal mean model and MAEE for the correlation structure for empirical power and 2000 data replicates are generated for empirical test size. Following Li et al.,^10^ both two-sided t-tests and z-test for testing $H_{0}$: δ=0 are examinated, constructed from the use of five different variance estimators for δ, the model-based variance, BC0,^36^ BC1,^35^ BC2,^38^ and BC3.^39^ The nominal test size is fixed at 5%; empirical size between 4.0% and 6.0% is considered to be acceptable as these bounds correspond to the 95% confidence interval derived from a binomial model with p=0.05 and 2000 replicates. Similarly, empirical power that differs by at most 2.6% from the nominal value is considered to be in agreement with the predicted power with 1000 simulations, which is derived as the margin of error from the 95% confidence interval for a binomial model with p=0.8 and 1000 replicates.

### 5.1 Type I error results for binary outcomes

**Figure 2.**
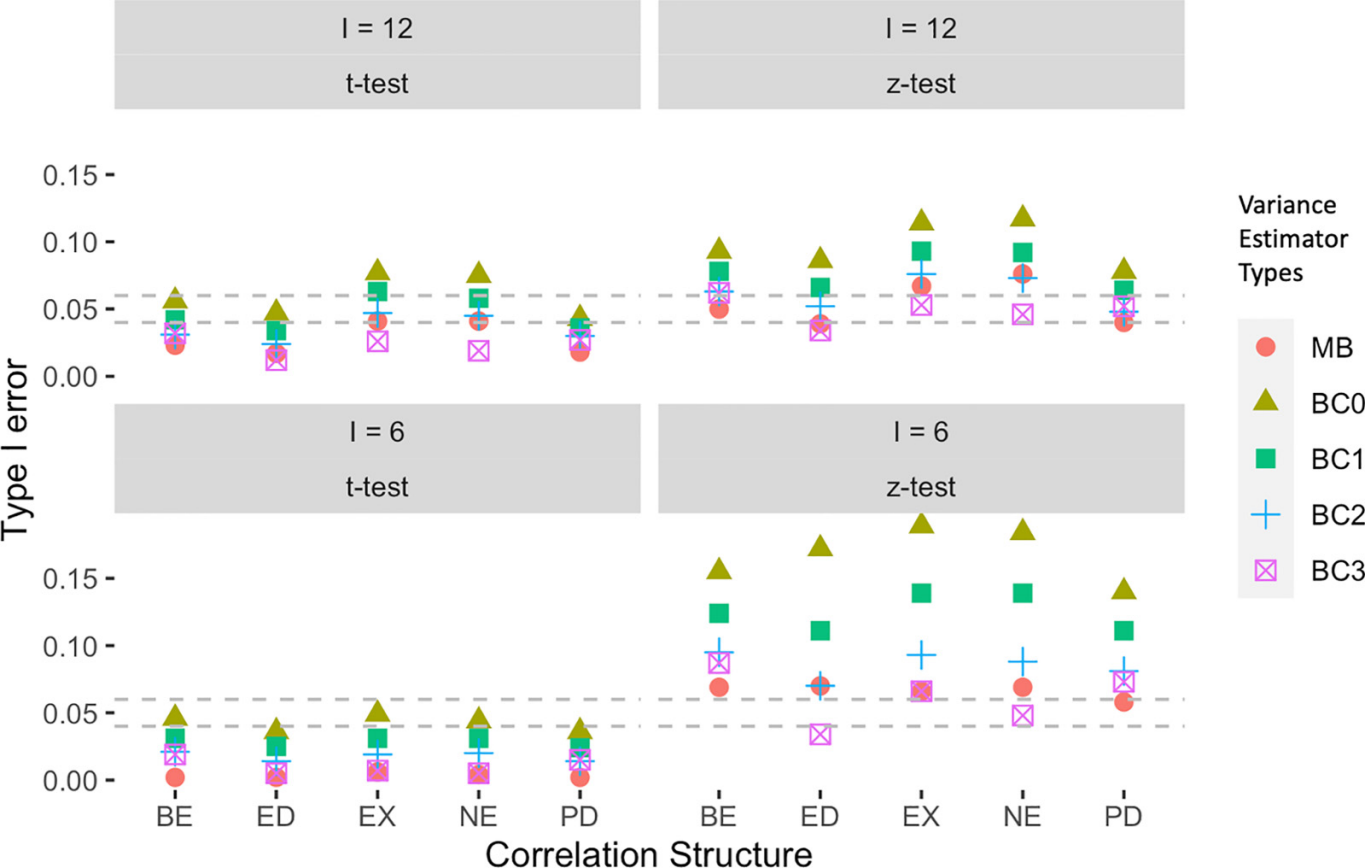
Empirical Type I error for binary responses under average intervention effects model. The gray lines indicated an acceptable boundary [4.0%, 6.0%] for the empirical test size compared to nominal test size, 0.05. Correlation Structures: EX: exchangeable correlation, ED: Exponential decay, NE: Nested exchangeable correlation, BE: Block exchangeable correlation, PD: Proportional decay correlation. Variance estimator: MB: Model-based variance, BC0: uncorrected sandwich estimator of Liang and Zeger,^36^ BC1: Bias-corrected sandwich variance of Kauermann and Carroll,^35^ BC2: Bias-corrected sandwich variance of Mancl and DeRouen,^38^ BC3: Bias-corrected sandwich variance of Fay and Graubard.^39^

For binary responses in the average intervention effects model, the z-test gives more liberal test size than the t-test and, not surprisingly, the differences were especially pronounced with six clusters (Figure 2). When using the z-test, variance estimators BC0, BC1, and BC2 consistently give test size exceeding the nominal 0.05 level, whereas model-based variance (MB) and BC3 tended to give acceptable test size under the five correlation structures. While test sizes are closer to the nominal test size for 12 clusters, BC0 and BC1 still consistently give test size higher than 0.05, whereas BC2 gives acceptable test size under ED and PD correlation structures. On the other hand, for the t-tests with six clusters, all variances estimators with the exception of BC0 tend to give conservative test size; BC1 gives the least conservative test, thus more acceptable for use, compared to the remaining variance estimators. For 12 clusters, BC1 with the t-test gives acceptable type I error under every correlation structure. In contrast, BC0 tends to give liberal test sizes under EX and NE and have acceptable test size under other correlation structures; MB, BC2, BC3 give conservative test sizes in nearly all scenarios. The results for type I error of binary responses under incremental intervention effects in Web Appendix C were qualitatively similar.

### 5.2 Power results for binary outcomes

**Figure 3.**
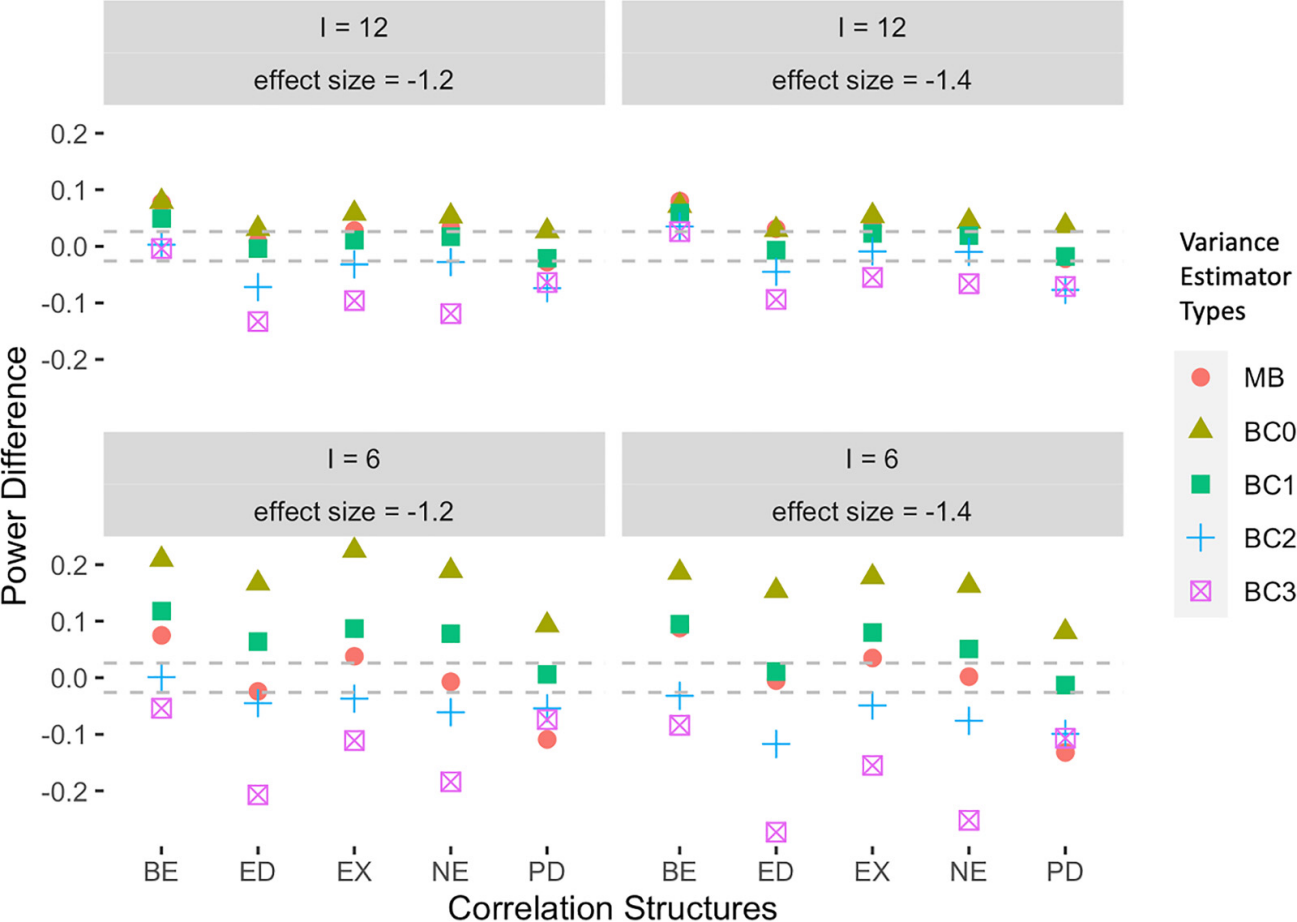
Power differences by simulated power minus the predicted power for binary responses under average intervention effects model with t-test. The gray lines indicated an acceptable boundary [ −2.6%, 2.6%]. Correlation Structures: EX: exchangeable correlation, ED: Exponential decay, NE: Nested exchangeable correlation, BE: Block exchangeable correlation, PD: Proportional decay correlation. Variance estimators: MB: Model-based variance, BC0: uncorrected sandwich estimator of Liang and Zeger,^36^ BC1: Bias-corrected sandwich variance of Kauermann and Carroll,^35^ BC2: Bias-corrected sandwich variance of Mancl and DeRouen,^38^ BC3: Bias-corrected sandwich variance of Fay and Graubard.^39^

The power results of binary responses under the average intervention effects model for 12 clusters show that, for two different effect sizes δ, the fast GEE power method based on the t-test accurately predicts empirical power based on the t-test with the BC1 variance estimator (Figure 3). In contrast, the conservatism of BC2 and BC3 is shown in their reduced empirical power in both absolute terms (Table 2) and relative to predicted power (Figure 3). In the case of six clusters, predicted power based on the t-test slightly underestimates the empirical power based on the t-test with the BC1 variance estimator. Predicted power based on the t-test closely matches the empirical power of model-based variance estimator for both six and 12 clusters and two effect sizes (Figure 3). This is not surprising because predicted power from the fast GEE power method is based on the model-based variance. The major drawback, of course, in the use of the MB variance is that GEE variance estimates are not consistent under misspecification of the correlation matrix.

**Table 2.** Empirical power of generalized estimating equations (GEE) analysis using different variance estimators and predicted power with average intervention effects model for binary responses based on the Connect-Home study.

			z-test				t-test				
I	Effect Size ${}^{a}$	Corr ${}^{b}$	Pred ${}^{c}$	MB ${}^{c}$	BC2 ${}^{c}$	BC3 ${}^{c}$	Pred ${}^{c}$	MB ${}^{c}$	BC0 ${}^{c}$	BC1 ${}^{c}$	BC2 ${}^{c}$
6	−1.2	EX	0.813	0.833	0.747	0.667	0.380	0.418	0.605	0.467	0.343
		ED	0.824	0.842	0.705	0.574	0.395	0.371	0.562	0.459	0.350
		NE	0.841	0.855	0.737	0.601	0.419	0.412	0.608	0.497	0.358
		BE	0.811	0.846	0.769	0.738	0.378	0.453	0.587	0.496	0.379
		PD	0.596	0.541	0.485	0.470	0.200	0.091	0.293	0.206	0.146
6	−1.4	EX	0.906	0.920	0.837	0.803	0.535	0.570	0.713	0.615	0.486
		ED	0.914	0.920	0.841	0.742	0.553	0.548	0.707	0.564	0.436
		NE	0.926	0.932	0.842	0.767	0.581	0.583	0.744	0.632	0.505
		BE	0.905	0.944	0.852	0.832	0.533	0.621	0.719	0.628	0.501
		PD	0.717	0.667	0.580	0.585	0.282	0.150	0.363	0.269	0.183
12	−1.2	EX	0.858	0.876	0.825	0.794	0.770	0.798	0.828	0.781	0.738
		ED	0.879	0.872	0.832	0.777	0.796	0.804	0.827	0.792	0.724
		NE	0.884	0.896	0.854	0.798	0.803	0.836	0.856	0.820	0.775
		BE	0.830	0.874	0.831	0.824	0.735	0.811	0.813	0.783	0.737
		PD	0.620	0.603	0.560	0.555	0.501	0.473	0.528	0.480	0.427
12	−1.4	EX	0.937	0.953	0.927	0.895	0.875	0.911	0.929	0.898	0.866
		ED	0.950	0.945	0.915	0.881	0.894	0.925	0.923	0.887	0.849
		NE	0.953	0.965	0.939	0.901	0.898	0.933	0.942	0.917	0.888
		BE	0.918	0.959	0.925	0.932	0.848	0.928	0.920	0.907	0.883
		PD	0.741	0.733	0.700	0.707	0.631	0.609	0.667	0.613	0.554

${}^{a}$Effect size δ.

${}^{b}$Correlation structures: EX: exchangeable correlation, α = (0.03, 0.03), ED: Exponential decay, α = (0.03, 0.8), NE: Nested exchangeable correlation, α = (0.03, 0.015), BE: Block exchangeable correlation, α = (0.03, 0.015, 0.2),PD: Proportional decay correlation, α = (0.03, 0.7).

${}^{c}$Pred: Predicted power, MB: Model-based variance, BC0: uncorrected sandwich estimator of Liang and Zeger,^36^ BC1: Bias-corrected sandwich variance of Kauermann and Carroll,^35^ BC2: Bias-corrected sandwich variance of Mancl and DeRouen,^38^ BC3: Bias-corrected sandwich variance of Fay and Graubard.^39^

While the sample size determination method should adequately predict the empirical power of the planned statistical analysis (including choice of variance estimator), the actual power of the study design and planned sample size as represented by empirical power is of paramount importance. In this regard, use of the t-tests results in a small to moderate loss of power relative to the z-test in the case of I=12 clusters, but a substantial loss in power for I=6 clusters owing to the use of cluster degrees of freedom, I−3. Table 2 only lists power for the variance estimators with acceptable empirical test-size as discussed in Section 5.1. As expected, power increases with an increase in the number of clusters and with an increase in the absolute magnitude of the effect size. Using the t-tests, predicted power is consistently similar to empirical power with BC1 and MB for 12 clusters (Figure 3 and Table 2). For six clusters, the predicted power is less than empirical power based on BC1 and closest to BC2 and MB. For the z-tests, predicted power is consistently closest to empirical power for the MB variance estimator. Power under PD correlation is the lowest among five correlation structures because of taking into account a substantial degree of decay in intra-individual correlation. These results are consistent with Li^29^ who found that the proportional decay correlation tended to have a greater estimated variance of intervention effects compared to that under the block exchangeable correlation of continuous responses. Results of statistical power for the incremental intervention effects model with binary responses are qualitatively similar to results for the average intervention effects model and are listed in Web Appendix C. Convergences rates are reported in Web Appendix E.

### 5.3 Results for continuous responses

For continuous responses (Web Appendix D), the type I error results under the average intervention effects model (Web Figure 3) also show that BC1 using the t-test, as well as BC3 and MB using the z-test perform consistently well compared to nominal test size. Under the incremental intervention effects model (Web Figure 5), BC1 using the t-test for six clusters always gives acceptable type I error, and shows less conservatism compared to the other scenarios. MB and BC3 still give an acceptable type I error using the z-test. Moreover, BC2 also gives acceptable type I error using the z-test with 12 clusters. In regards of the power results for continuous responses under the average intervention effects model (Web Figure 4 and Web Table 3), the fast GEE power method using the t-test in general accurately predicts the power by BC1, except for six clusters with the smaller test size δ=0.5. For the z-tests, predicted power is still consistently closest to empirical power by the MB variance estimator. Under the incremental intervention effects model (Web Figure 6 and Web Table 4), the fast GEE power method using the t-test gives the predicted power similar to BC1 for 12 clusters, and less than BC1 for six clusters. For the z-test, results are similar to the average intervention effects model.

## 6 Application of the fast GEE power method

### 6.1 Predicted power of six different SW-CRT designs

The fast GEE power calculation method is applied to compare power among six SW-CRTs designs (Web Appendix F) based on the Connect-Home trial for continuous and binary outcomes. The purpose of this exercise is to illustrate how the fast power method may be used to compare power among several complete and incomplete stepped wedge designs under consideration during study planning. Each design has a fixed total of 360 patients, six treatment sequences and from 16 to 22 periods. Design A is the incomplete SW-CRT for Connect-Home trial and Design F is a complete SW-CRT. The other four designs show alternative incomplete SW-CRTs and vary according to the length of implementation periods, the use of staggered study entry and termination of clusters. Each study includes six or 12 clusters or SNFs with four or two patients per cluster-period, respectively, so that the total number of patients is always 360. As in the simulation study, the power using the t-test for binary responses are evaluated with intervention effect size fixed at δ=−1.2 for the average intervention effects model and δ=−2.4 for the incremental intervention effects model with q=10 and a gently decreasing linear period effects such that $\beta_{0}=$ 0.85 and $\beta_{1}=-0.01$. Meanwhile, power using the t-test for continuous responses is evaluated with intervention effect size fixed at δ=0.5 for the average intervention effects model and δ=1.0 for the incremental intervention effects model also with q=10 and a gently decreasing linear period effects such that $\beta_{0}=2$ and $\beta_{1}=-0.05$. Power is assessed under the three correlation structures for cross-sectional designs in Table 1 with the same correlation parameters as in the simulation section.

For binary responses, power with 6 clusters and a cluster-period size of 4 is always much smaller than 12 clusters and a cluster-period size of 2 because power of the t-test is especially affected by the number of clusters (Table 3). Thus, 12 clusters are recommended to achieve power more than 80% for the binary responses for all scenarios. Among the six SW-CRT designs under the average intervention effects model, Design C and Design F, which have no implementation periods, have the greatest power. The power of Designs B and E are in the middle level but still greater than those of Designs A and D, respectively. In summary, incompleteness due to implementation periods causes greater loss of power compared to staggered study entry and termination. For a given study design, the impact of correlation structure is relatively mild. Power under the nested exchangeable correlation structure is always the greatest, whereas the relationship between power under the exchangeable and exponential decay correlation structures is not consistent. The exponential decay correlation tends to have greater power than the exchangeable correlation, except for the Designs D/E/F with six clusters.

**Table 3.** The predicted GEE power of the t-test for binary responses in cross-sectional SW-CRTs having six sequences, 16 to 22 periods, and a total of 360 participants with a maximum of four participants per cluster-period.

Model ${}^{b}$	I	Corr ${}^{c}$	A ${}^{a}$	B ${}^{a}$	C ${}^{a}$	D ${}^{a}$	E ${}^{a}$	F ${}^{a}$
Staggered entry/termination			Yes	Yes	Yes	No	No	No
Number of implementation periods			2	1	0	2	1	0
Average	6	EX	0.380	0.467	0.568	0.419	0.505	0.599
		ED	0.395	0.481	0.582	0.386	0.474	0.578
		NE	0.419	0.508	0.607	0.436	0.524	0.619
	12	EX	0.770	0.834	0.888	0.785	0.844	0.894
		ED	0.796	0.853	0.902	0.786	0.847	0.899
		NE	0.803	0.860	0.907	0.804	0.861	0.908
Incremental	6	EX	0.495	0.526	0.568	0.623	0.647	0.681
		ED	0.543	0.575	0.615	0.636	0.663	0.698
		NE	0.560	0.598	0.645	0.675	0.705	0.741
	12	EX	0.863	0.882	0.904	0.917	0.929	0.942
		ED	0.893	0.910	0.927	0.933	0.943	0.955
		NE	0.894	0.912	0.931	0.937	0.948	0.959

${}^{a}$A–F: Designs A to F are shown in Appendix F for six clusters. For 12 clusters, the cluster-period sizes are half the amount shown, that is, 1 and 2 instead of 2 and 4.

${}^{b}$Marginal models: Average: average intervention effects model with δ=−1.2, Incremental: incremental intervention effects model with δ=−2.4 and maximum intervention periods equaling to 10.

${}^{c}$Correlation structures: EX: exchangeable correlation, α = (0.03, 0.03), ED: Exponential decay, α = (0.03, 0.8), NE: Nested exchangeable correlation, α = (0.03, 0.015).

Comparative results for power for binary responses under the six designs is notably different for the incremental intervention effects model. The power of Designs D/E/F is consistently greater than those of Designs A/B/C, respectively. Thus, the introduction of staggered study entry and termination of clusters into the stepped wedge designs has a greater negative impact on power than the introduction of implementation periods under the incremental interventions effects model. While this result is the opposite from what was observed under the average interventions effects model, power under both marginal means models was always best under the complete design F, and almost always worst under Design A that used staggered entry/termination and two implementation periods. The pattern of GEE power with respect to the correlation structures was similar for the two marginal mean models. Finally, the results for continuous responses (Web Table 7) align well with those for binary responses. All power results in this section and the next were produced by the SAS macro **CRTFASTGEEPWR**. Descriptions of the inputs and outputs of the SAS macro are provided in Section G of the Web Appendix along with three examples of its application to incomplete and complete SW-CRTs.

### 6.2 Sample size calculation for the Connect-Home trial

Although Design A was the overall worst performer in terms of power among the six study designs in Section 6.1., it was nonetheless chosen for the Connect-Home trial because, as noted in Section 2., logistical considerations dictated both staggered entry/termination and two implementation periods. Results of the simulation study and the application of the fast GEE power method in Table 3 show that predicted power using the t-test for incomplete Design A with six clusters is always below 80%. Thus, this section explores how the cluster-period size impacts predicted power for the Connect-Home trial and determines the suitable sample size in order to reach 80% power for binary and continuous outcomes. Specifically, Design A of the Connect-Home trial (Figure 1) is considered with a constant cluster-period size, ranging from 4 to 9. Two marginal models, each with two effect sizes are considered in the sample size calculation for binary and continuous responses. As in the simulation study, the power using the t-test for binary responses is evaluated with intervention effect size fixed at δ=−1.2 and δ=−1.4 for the average intervention effects model, and δ=−1.8 and δ=−2.4 for the incremental intervention effects model with q=10 and a gently decreasing linear period effects such that $\beta_{0}=$ 0.85 and $\beta_{1}=-0.01$. Meanwhile, predicted power using the t-test for continuous responses is evaluated with intervention effect size fixed at δ=0.5 and δ=0.7 for the average intervention effects model, and δ=0.8 and δ=1.0 for the incremental intervention effects model also with q=10 and a gently decreasing linear period effects such that $\beta_{0}=2$ and $\beta_{1}=-0.05$. The three cross-sectional design marginal correlation structures in Table 1 are analyzed with the same correlation parameters as in the simulation section.

From Table 4 for binary responses, predicted power increases incrementally with increasing cluster-period size. Under the average intervention effects model, predicted power reaches 80% under all the three correlation structures when there are 8 or more patients in each cluster-period with an effect size of −1.4. Meanwhile, for the incremental intervention effects model, predicted power reaches 80% when there are nine patients in each cluster-period with an effect size of −2.4. Regarding the power comparison between the correlation structures, nested exchangeable always gives the greatest predicted power among three correlation structures. Moreover, under the nested exchangeable correlation structure, the cluster-period size could be reduced to seven patients under the average intervention effects model with effect size of −1.4 and eight patients under the incremental intervention effects model with effect size of −2.4 for 80% power. As cluster-period size increases, the power under exchangeable correlation structure ultimately exceeds the power under exponential decay and becomes closer to the power under nested exchangeable correlation structure. For continuous responses, when the cluster-period size is seven or more, predicted power reaches 80% under the average intervention effects model with effect size of 0.7. The relationship between three correlation structures is the same as with binary responses (Web Table 8).

**Table 4.** Fast GEE power using t-test under different cluster-period size for Connect-Home trial with six clusters for binary responses.

			Cluster-period size ${}^{d}$					
Model ${}^{a}$	Effect size ${}^{b}$	Corr ${}^{c}$	4	5	6	7	8	9
Average	−1.2	EX	0.380	0.478	0.567	0.644	0.709	0.761
		ED	0.395	0.479	0.551	0.611	0.660	0.700
		NE	0.419	0.516	0.599	0.667	0.722	0.765
Average	−1.4	EX	0.535	0.645	0.731	0.794	0.840	0.874
		ED	0.553	0.647	0.716	0.767	0.805	0.834
		NE	0.581	0.683	0.757	0.810	0.848	0.876
Incremental	−1.8	EX	0.275	0.343	0.411	0.478	0.540	0.570
		ED	0.305	0.369	0.427	0.478	0.521	0.559
		NE	0.316	0.390	0.460	0.522	0.578	0.626
Incremental	−2.4	EX	0.495	0.598	0.682	0.747	0.798	0.836
		ED	0.543	0.632	0.698	0.747	0.784	0.812
		NE	0.560	0.659	0.732	0.785	0.825	0.855

${}^{a}$Marginal models: Average: average intervention effects model, Incremental: incremental intervention effects model.

${}^{b}$Effect size: δ.

${}^{c}$Correlation structures: EX: exchangeable correlation, α = (0.03,0.03), ED: Exponential decay, α = (0.03,0.8), NE: Nested exchangeable correlation, α = (0.03,0.015).

${}^{d}$Cluster-period size: the number of participants in a cluster-period.

## 7 Discussion

This article proposes a fast GEE power method for binary and continuous responses in complete and incomplete stepped wedge cluster randomized trials. Extensive simulation studies demonstrate that the fast GEE power method can reliably predict power in real-world cross-sectional and closed-cohort incomplete stepped wedge trials when the statistical analysis is based upon GEE with bias-corrected sandwich variance estimators. The fast GEE power approach was illustrated in the planning of an incomplete stepped wedge design for Connect-Home, an intervention to improve outcomes for rehabilitation patients transitioning from skilled nursing facilities to home care. A SAS macro CRTFASTGEEPWR was developed to implement the power method.

This study is novel in several aspects. Through specification of the “Design Pattern” matrix and “Completeness Matrix” in the spirit of Hemming et al.^3^ and Rochon,^40^ the general GEE power method is implemented for complete and incomplete stepped wedge designs, thereby extending the power methods for complete designs of Li et al.^10^ to designs with staggered enrollment of clusters, implementation periods, and planned early withdrawal of clusters, as applications shown in Section 6. Indeed, the lack of closed-form formula for non-continuous outcomes motivates the fast, numerical approach for power calculation. While this article focuses on linear time trends in the marginal mean model based on the Connect-Home trial, our approach accommodates categorical and any other time trends. Moreover, the SAS macro CRTFASTGEEPWR is developed to calculate power for binary, count, and continuous outcomes with linear or categorical periods effects based complete or incomplete CRTs. Recently, Ouyang et al.^43^ reviewed 18 power calculators for SW-CRTs based on GLMMs or GEEs and report that four calculators^24,44,30,41^ which may cover most scenarios of interest, including our SAS macro CRTFASTGEEPWR. Moreover, there is no other single article that unifies the five typical correlation models in an approach for designing cross-sectional and closed-cohort SW-CRTs. With respect to the statistical analysis, this is the first paper that investigates the small-sample performance of GEE under incomplete designs with respect to type I error rate and power. Finally, the log-linear correlation estimating equations with finite-sample corrections for ED and PD correlation models with individual-level data are novel for the SW-CRT applications.

In addition to the focus on incomplete stepped wedge designs, this article builds upon Li et al.^10^ who restrict attention to the average intervention effects model for continuous and binary responses with categorical period fixed effects and an exchangeable or nested exchangeable correlation structure for complete cross-sectional stepped wedge designs. In the current work, the specification of linear period effects allows use of the t-test for a SW-CRT when there are more follow-up periods than clusters, based on the conventional definition of degrees of freedom as equal to the number of clusters minus the number of marginal mean parameters.^45^ Additionally, the present article considers two distinct marginal mean models, the popular average intervention effects model and the incremental intervention effects model. The latter model allows for a gradual uptake of intervention across periods which is similar to the linear-time-on-treatment effect representation within the linear mixed model framework.^5,33^

The simulation studies support the use of the t-test with BC1 variance estimator, which always gives the closest (or acceptably conservative) empirical test size relative to the nominal value (0.05) with six or twelves clusters. We summarized the key simulation settings and results from different articles^12,10,46,29,11,18,17^ with GEE analysis for SW-CRTs under a small number of clusters in Web Table 11. According to Web Table 11, the majority of studies advised using the t-test with the BC1 variance estimator to control type I error or bias of intervention estimations. Note that, in complete SW-CRTs, Ford and Westgate^17^ recently demonstrated that the average of BC1 and BC2 standard errors could best maintain valid type I error rate; however, the empirical power of their bias-correction remains unexplored. Future work is needed to study whether their average standard error estimator provides empirical power close to a prediction formula such as proposed here under incomplete SW-CRTs.

Importantly, the analytical power by the fast GEE power method agrees well with simulated power in complete and incomplete SW-CRTs having twelve clusters with cluster-period sizes of two when data are analyzed using GEE with the bias-corrected sandwich variance estimator BC1. For designs of six clusters with cluster-period sizes of four, the analytical power using t-test is similar to the simulated power with correctly specified model-based variance estimator and is more conservative compared to the power with BC1 variance estimator. Thus, the results support the use of fast GEE power methods with t-test for 12 clusters and extend the minimal number of clusters to have valid simulated GEE power with BC1 variance estimator from eight clusters in Li et al.^10^ to six clusters. However, performance results for BC3 in the current article are poorer compared to Li et al.^10^ and Scott et al.^12^ The different performance of BC3 could be related to the incomplete design characteristics of the Connect-Home trial. Scott et al.^12^ and Thompson et al.^18^ showed that the BC3 tended to have a similar empirical power with BC1 under the complete SW-CRT. Notably, BC3 is constructed by a diagonal matrix for finite-sample correction that might be more easily affected by the information loss and have greater variances due to the missingness under the incomplete SW-CRTs compared to BC0, BC1, and BC2. In short, our results lead to a recommendation not to use the BC3 correction for incomplete stepped wedge designs with a small number of clusters.

In assessing the validity of the fast GEE power method, its predicted power was compared to simulated power using statistical analysis based on GEE with MAEE finite-sample adjustment for correlation estimates. Estimation of correlation parameters by MAEE, which was not a focus of the current work, has been shown to substantially reduce bias and improve coverage for intraclass correlations in clustered randomized trials including those with stepped wedge designs.^19^ As discussed in Section 1.2, marginal mean modeling facilitates the estimation and reporting of ICC estimates in cluster randomized trials, as recommended by the CONSORT statement for stepped wedge trials.^47^ While the use of MAEE produces better ICC estimates, bias-correction for the ICC estimates has been found to have negligible impact on the analysis of the intervention effect in marginal mean models for cluster randomized trials.^48^

Nevertheless, we further extended the MAEE to accommodate the exponential decay and proportional decay correlation structure for cross-sectional and closed-cohort, respectively, by using the log link in the generalized linear model for the correlation parameters. However, the convergence rate of estimation results under exponential decay and proportional decay correlation were lower than the other correlation structures (Web Appendix E). Because the simulation study was motivated by the Connect-Home SW-CRT, which has six clusters, the relatively low convergence rate was not unexpected. With a higher number of clusters, the convergence rate increases for ED but not for PD. For convergence failures under decay correlation structures, the estimation of correlations out of their natural boundary and non-convergence under Newton–Raphson method are the main causes. In order to lessen the chance of a convergence failure, the range of feasible correlation parameters in terms of positive-definite correlation matrices should be checked in the design phase of SW-CRTs. Future work could be done to improve the convergence rate under decaying correlation structures with the GEE/MAEE procedure for small numbers of clusters, such as replacing Newton–Raphson method with the quasi-least squares approach in Li.^29^

The proposed fast GEE power method was applied in a hypothetical study planning scenario where six prospective stepped wedge designs were compared with respect to their power given a fixed number of clusters and total sample size. This demonstration enabled an assessment of the impact on power of certain features of incomplete stepped wedge designs. While the relative impact of staggered enrollment and implementation periods depended upon the assumptions of the marginal mean model and within-cluster correlation structure, the general finding suggested that the use of incomplete SW-CRT designs decreases power relative to comparable complete designs. While power tended to be the greatest for the nested exchangeable correlation matrix among the five correlation structures studied, this finding may not apply to other settings such as SW-CRTs with large cluster-period sizes. Importantly, the choice of correlation structure in power analysis should be based on the study design, population and outcomes, for example, cross-sectional versus closed-cohort design and whether correlation decay is expected based on trial duration.

In conclusion, the fast GEE power method provides a computationally efficient, non-simulation approach to determine the power and sample size of complete and incomplete SW-CRTs. Both the fast GEE power method and the GEE/MAEE analysis procedure can also be applied to count responses and to marginal models with individual level covariates. For example, open-cohort studies are a possible extension of the methods, without the restriction of a constant number of patients across periods and allowing for missing observations common in real-life data analysis. Finally, the procedures could also be adapted to the design and analysis of various types of complete and incomplete parallel trials, including staggered incomplete parallel CRTs^3^ and cluster crossover trials.^46^

## Supplemental Material

sj-pdf-1-smm-10.1177_09622802221129861 - Supplemental material for A general method for calculating power for GEE analysis of complete and incomplete stepped wedge cluster randomized trials Click here for additional data file. Supplemental material, sj-pdf-1-smm-10.1177_09622802221129861 for A general method for calculating power for GEE analysis of complete and incomplete stepped wedge cluster randomized trials by Ying Zhang, John S Preisser, Elizabeth L Turner, Paul J Rathouz, Mark Toles and Fan Li in Statistical Methods in Medical Research
