# Supplementary material for: A general method for calculating power for GEE analysis of complete and incomplete stepped wedge cluster randomized trials
Source: Stat Methods Med Res. 2022 Oct 17;32(1):71–87. doi: 10.1177/09622802221129861 (PMC9814029; doi:10.1177/09622802221129861)
Supplement: sj-pdf-1-smm-10.1177_09622802221129861 - Supplemental material for A general method for calculating power for GEE analysis of complete and incomplete stepped wedge cluster randomized trials [file sj-pdf-1-smm-10.1177_09622802221129861.pdf]

Statistical Methods in Medical Research
Appendix 1–26
©The Author(s) 2022
Reprints and permission:
sagepub.co.uk/journalsPermissions.nav
DOI: 10.1177/09622802221129861
journals.sagepub.com/home/smm
SAGE

Web appendix for ”A general method for calculating power for GEE analysis of complete and incomplete stepped wedge cluster randomized trials”

Ying Zhang*, John S. Preisser, Elizabeth L. Turner , Paul J. Rathouz , Mark Toles and Fan Li

Corresponding author:
Ying Zhang, zying@live.unc.edu

[Version:]

A. The Design Pattern Matrix and Completeness Matrix of the Connect-Home study

Web Table 1. The detailed stepped wedge design specifications of the Connect-Home trial in Figure 1 and Section 3.1

i^a	${b_{i0}}^b$	${b_{i1}}^b$	${b_i}^b$	${q_{i0}}^c$	${q_{i1}}^c$	${q_i}^c$
1	1	5	5	8	17	10
2	2	7	6	10	18	9
3	3	9	7	12	19	8
4	4	11	8	14	20	7
5	5	13	9	16	21	6
6	6	15	10	18	22	5

a: The ith cluster in the sth sequence, assuming one cluster in each sequence

b: b_{i0}, b_{i1} are the first and the last period of the control condition; b_i is the number of periods in the control condition in the ith cluster.

c: q_{i0}, q_{i1} are the first and the last period of the intervention condition; q_i is the number of periods in the intervention condition in the ith cluster.

Correspondingly, the Design Pattern Matrix (**DP**) and Completeness Matrix (**CM**) of the Connect-Home trial are:

$$\mathbf{DP} = \begin{pmatrix} 0 & 0 & 0 & 0 & 0 & . & . & 1 & 1 & 1 & 1 & 1 & 1 & 1 & 1 & 1 & 1 & . & . & . & . & . \\ . & 0 & 0 & 0 & 0 & 0 & 0 & . & . & 1 & 1 & 1 & 1 & 1 & 1 & 1 & 1 & 1 & . & . & . & . \\ . & . & 0 & 0 & 0 & 0 & 0 & 0 & 0 & . & . & 1 & 1 & 1 & 1 & 1 & 1 & 1 & 1 & . & . & . \\ . & . & . & 0 & 0 & 0 & 0 & 0 & 0 & 0 & 0 & . & . & 1 & 1 & 1 & 1 & 1 & 1 & 1 & . & . \\ . & . & . & . & 0 & 0 & 0 & 0 & 0 & 0 & 0 & 0 & 0 & . & . & 1 & 1 & 1 & 1 & 1 & 1 & . \\ . & . & . & . & . & 0 & 0 & 0 & 0 & 0 & 0 & 0 & 0 & 0 & 0 & . & . & 1 & 1 & 1 & 1 & 1 \end{pmatrix}$$

$$\mathbf{CM} = \begin{pmatrix} 1 & 1 & 1 & 1 & 1 & 0 & 0 & 1 & 1 & 1 & 1 & 1 & 1 & 1 & 1 & 1 & 1 & 0 & 0 & 0 & 0 & 0 \\ 0 & 1 & 1 & 1 & 1 & 1 & 1 & 0 & 0 & 1 & 1 & 1 & 1 & 1 & 1 & 1 & 1 & 1 & 0 & 0 & 0 & 0 \\ 0 & 0 & 1 & 1 & 1 & 1 & 1 & 1 & 1 & 0 & 0 & 1 & 1 & 1 & 1 & 1 & 1 & 1 & 1 & 0 & 0 & 0 \\ 0 & 0 & 0 & 1 & 1 & 1 & 1 & 1 & 1 & 1 & 1 & 0 & 0 & 1 & 1 & 1 & 1 & 1 & 1 & 1 & 0 & 0 \\ 0 & 0 & 0 & 0 & 1 & 1 & 1 & 1 & 1 & 1 & 1 & 1 & 1 & 0 & 0 & 1 & 1 & 1 & 1 & 1 & 1 & 0 \\ 0 & 0 & 0 & 0 & 0 & 1 & 1 & 1 & 1 & 1 & 1 & 1 & 1 & 1 & 1 & 0 & 0 & 1 & 1 & 1 & 1 & 1 \end{pmatrix}$$

B. The examples of five correlation structures

For a SW-CRT with 4 periods and 2 individuals in each period, the five correlation structures are listed here for illustration. For the responses vector $\boldsymbol{y}_i = (y_{i11}, y_{i12}, y_{i21}, y_{i22}, y_{i31}, y_{i32}, y_{i41}, y_{i42})$.
Under the exchangeable structure with ICCs = (α_0)

$$\boldsymbol{R} = \begin{pmatrix} 1 & \alpha_0 & \alpha_0 & \alpha_0 & \alpha_0 & \alpha_0 & \alpha_0 & \alpha_0 \\ \alpha_0 & 1 & \alpha_0 & \alpha_0 & \alpha_0 & \alpha_0 & \alpha_0 & \alpha_0 \\ \alpha_0 & \alpha_0 & 1 & \alpha_0 & \alpha_0 & \alpha_0 & \alpha_0 & \alpha_0 \\ \alpha_0 & \alpha_0 & \alpha_0 & 1 & \alpha_0 & \alpha_0 & \alpha_0 & \alpha_0 \\ \alpha_0 & \alpha_0 & \alpha_0 & \alpha_0 & 1 & \alpha_0 & \alpha_0 & \alpha_0 \\ \alpha_0 & \alpha_0 & \alpha_0 & \alpha_0 & \alpha_0 & 1 & \alpha_0 & \alpha_0 \\ \alpha_0 & \alpha_0 & \alpha_0 & \alpha_0 & \alpha_0 & \alpha_0 & 1 & \alpha_0 \\ \alpha_0 & \alpha_0 & \alpha_0 & \alpha_0 & \alpha_0 & \alpha_0 & \alpha_0 & 1 \end{pmatrix}$$

Under the nested exchangeable correlation structure with ICCs = (α_0, α_1)

$$\boldsymbol{R} = \begin{pmatrix} 1 & \alpha_0 & \alpha_1 & \alpha_1 & \alpha_1 & \alpha_1 & \alpha_1 & \alpha_1 \\ \alpha_0 & 1 & \alpha_1 & \alpha_1 & \alpha_1 & \alpha_1 & \alpha_1 & \alpha_1 \\ \alpha_1 & \alpha_1 & 1 & \alpha_0 & \alpha_1 & \alpha_1 & \alpha_1 & \alpha_1 \\ \alpha_1 & \alpha_1 & \alpha_0 & 1 & \alpha_1 & \alpha_1 & \alpha_1 & \alpha_1 \\ \alpha_1 & \alpha_1 & \alpha_1 & \alpha_1 & 1 & \alpha_0 & \alpha_1 & \alpha_1 \\ \alpha_1 & \alpha_1 & \alpha_1 & \alpha_1 & \alpha_0 & 1 & \alpha_1 & \alpha_1 \\ \alpha_1 & \alpha_1 & \alpha_1 & \alpha_1 & \alpha_1 & \alpha_1 & 1 & \alpha_0 \\ \alpha_1 & \alpha_1 & \alpha_1 & \alpha_1 & \alpha_1 & \alpha_1 & \alpha_0 & 1 \end{pmatrix}$$

Under the exponential decay correlation structure with ICCs = (α_0, ρ)

$$\boldsymbol{R} = \begin{pmatrix} 1 & \alpha_0 & \alpha_0\rho & \alpha_0\rho & \alpha_0\rho^2 & \alpha_0\rho^2 & \alpha_0\rho^3 & \alpha_0\rho^3 \\ \alpha_0 & 1 & \alpha_0\rho & \alpha_0\rho & \alpha_0\rho^2 & \alpha_0\rho^2 & \alpha_0\rho^3 & \alpha_0\rho^3 \\ \alpha_0\rho & \alpha_0\rho & 1 & \alpha_0 & \alpha_0\rho & \alpha_0\rho & \alpha_0\rho^2 & \alpha_0\rho^2 \\ \alpha_0\rho & \alpha_0\rho & \alpha_0 & 1 & \alpha_0\rho & \alpha_0\rho & \alpha_0\rho^2 & \alpha_0\rho^2 \\ \alpha_0\rho^2 & \alpha_0\rho^2 & \alpha_0\rho & \alpha_0\rho & 1 & \alpha_0 & \alpha_0\rho & \alpha_0\rho \\ \alpha_0\rho^2 & \alpha_0\rho^2 & \alpha_0\rho & \alpha_0\rho & \alpha_0 & 1 & \alpha_0\rho & \alpha_0\rho \\ \alpha_0\rho^3 & \alpha_0\rho^3 & \alpha_0\rho^2 & \alpha_0\rho^2 & \alpha_0\rho & \alpha_0\rho & 1 & \alpha_0 \\ \alpha_0\rho^3 & \alpha_0\rho^3 & \alpha_0\rho^2 & \alpha_0\rho^2 & \alpha_0\rho & \alpha_0\rho & \alpha_0 & 1 \end{pmatrix}$$

Under the block exchangeable correlation structure with ICCs = $(\alpha_0, \alpha_1, \alpha_2)$

$$\boldsymbol{R} = \begin{pmatrix} 1 & \alpha_0 & \alpha_2 & \alpha_1 & \alpha_2 & \alpha_1 & \alpha_2 & \alpha_1 \\ \alpha_0 & 1 & \alpha_1 & \alpha_2 & \alpha_1 & \alpha_2 & \alpha_1 & \alpha_2 \\ \alpha_2 & \alpha_1 & 1 & \alpha_0 & \alpha_2 & \alpha_1 & \alpha_2 & \alpha_1 \\ \alpha_1 & \alpha_2 & \alpha_0 & 1 & \alpha_1 & \alpha_2 & \alpha_1 & \alpha_2 \\ \alpha_2 & \alpha_1 & \alpha_2 & \alpha_1 & 1 & \alpha_0 & \alpha_2 & \alpha_1 \\ \alpha_1 & \alpha_2 & \alpha_1 & \alpha_2 & \alpha_0 & 1 & \alpha_1 & \alpha_2 \\ \alpha_2 & \alpha_1 & \alpha_2 & \alpha_1 & \alpha_2 & \alpha_1 & 1 & \alpha_0 \\ \alpha_1 & \alpha_2 & \alpha_1 & \alpha_2 & \alpha_1 & \alpha_2 & \alpha_0 & 1 \end{pmatrix}$$

Under the proportional decay correlation structure with ICCs = (α_0, ρ)

$$\boldsymbol{R} = \begin{pmatrix} 1 & \alpha_0 & \rho & \alpha_0\rho & \rho^2 & \alpha_0\rho^2 & \rho^3 & \alpha_0\rho^3 \\ \alpha_0 & 1 & \alpha_0\rho & \rho & \alpha_0\rho^2 & \rho^2 & \alpha_0\rho^3 & \rho^3 \\ \rho & \alpha_0\rho & 1 & \alpha_0 & \rho & \alpha_0\rho & \rho^2 & \alpha_0\rho^2 \\ \alpha_0\rho & \rho & \alpha_0 & 1 & \alpha_0\rho & \rho & \alpha_0\rho^2 & \rho^2 \\ \rho^2 & \alpha_0\rho^2 & \rho & \alpha_0\rho & 1 & \alpha_0 & \rho & \alpha_0\rho \\ \alpha_0\rho^2 & \rho^2 & \alpha_0\rho & \rho & \alpha_0 & 1 & \alpha_0\rho & \rho \\ \rho^3 & \alpha_0\rho^3 & \rho^2 & \alpha_0\rho^2 & \rho & \alpha_0\rho & 1 & \alpha_0 \\ \alpha_0\rho^3 & \rho^3 & \alpha_0\rho^2 & \rho^2 & \alpha_0\rho & \rho & \alpha_0 & 1 \end{pmatrix}$$

C. Simulated results for binary responses under incremental intervention effects model

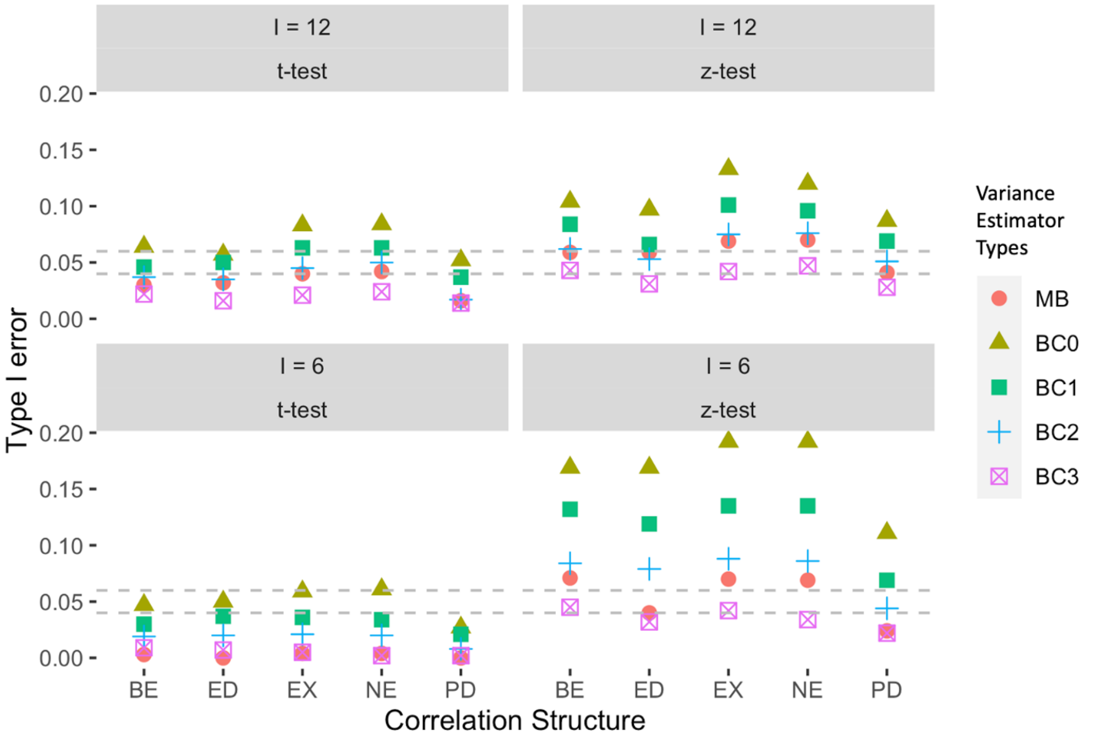

Web Figure 1. Type I error under incremental intervention effects model for binary responses. The grey lines indicated an acceptable boundary [4.0%,6.0%] for the empirical test size compared to nominal test size, 0.05. Correlation structures: EX: Exchangeable correlation, ED: Exponential decay correlation,NE: Nested exchangeable correlation,,BE: Block exchangeable correlation,PD: Proportional decay correlation. MB: Model-based variance, BC0: uncorrected sandwich estimator of Liang and Zeger (1986), BC1:Bias-corrected sandwich variance of Kauermann and Carroll (2001), BC2: Bias-corrected sandwich variance of Mancl and DeRouen (2001), BC3: Bias-corrected sandwich variance of Fay and Graubard (2001).

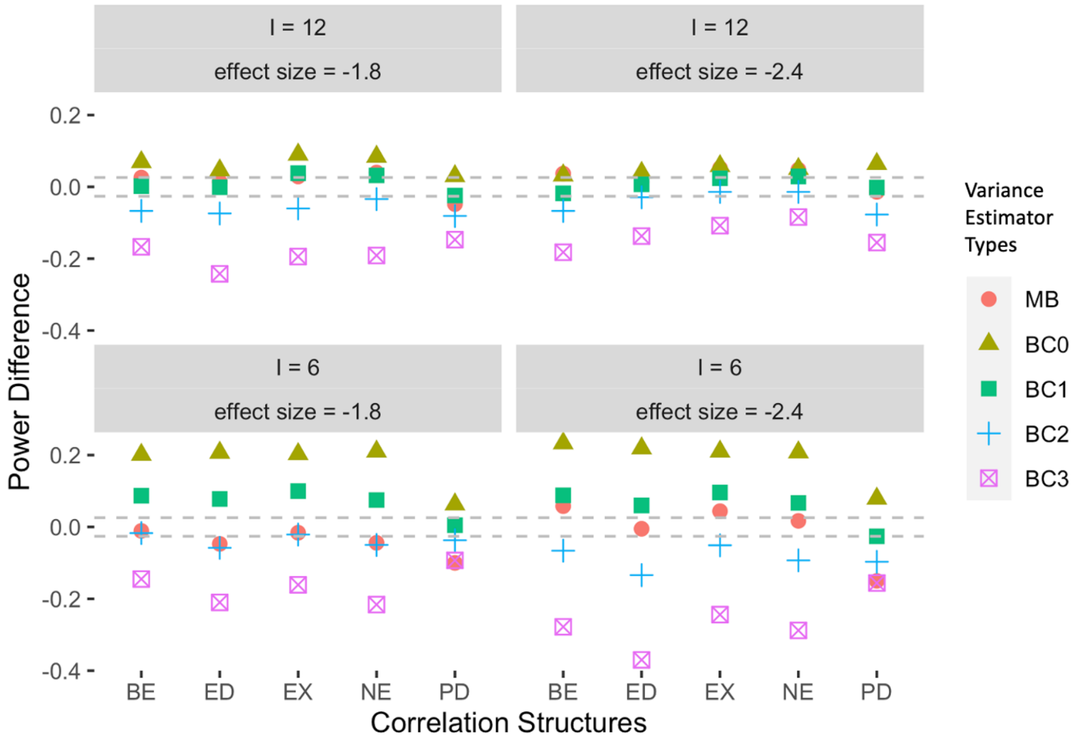

Web Figure 2. Power differences by simulated power minus the predicted power for binary responses under incremental intervention effects model with t-test. EX: Exchangeable correlation, ED: Exponential decay correlation,NE: Nested exchangeable correlation,BE: Block exchangeable correlation,PD: Proportional decay correlation.MB: Model-based variance, BC0: uncorrected sandwich estimator of Liang and Zeger (1986), BC1:Bias-corrected sandwich variance of Kauermann and Carroll (2001), BC2: Bias-corrected sandwich variance of Mancl and DeRouen (2001), BC3: Bias-corrected sandwich variance of Fay and Graubard (2001).

Web Table 2. Empirical power of GEE analysis using different variance estimators and predicted power with incremental intervention effects model for binary responses.

			z-test				t-test				
**I**	**Effect Size**[a]	**Corr**[b]	**Pred**[c]	**MB**[c]	**BC2**[c]	**BC3**[c]	**Pred**[c]	**MB**[c]	**BC0**[c]	**BC1**[c]	**BC2**[c]
6	-1.8	EX	0.709	0.720	0.610	0.386	0.275	0.259	0.478	0.375	0.254
		ED	0.744	0.722	0.61	0.349	0.305	0.258	0.512	0.383	0.247
		NE	0.755	0.762	0.631	0.385	0.316	0.272	0.526	0.391	0.266
		BE	0.690	0.690	0.577	0.371	0.260	0.249	0.461	0.347	0.243
		PD	0.437	0.365	0.314	0.189	0.131	0.031	0.194	0.136	0.094
6	-2.4	EX	0.887	0.903	0.820	0.627	0.495	0.539	0.705	0.591	0.444
		ED	0.910	0.919	0.854	0.625	0.542	0.537	0.761	0.602	0.408
		NE	0.917	0.928	0.843	0.624	0.560	0.577	0.768	0.627	0.467
		BE	0.872	0.905	0.784	0.549	0.468	0.526	0.701	0.556	0.402
		PD	0.602	0.577	0.466	0.286	0.214	0.064	0.293	0.188	0.117
12	-1.8	EX	0.775	0.805	0.747	0.614	0.669	0.698	0.759	0.707	0.609
		ED	0.814	0.790	0.742	0.618	0.716	0.730	0.762	0.715	0.642
		NE	0.815	0.838	0.775	0.663	0.717	0.757	0.801	0.749	0.683
		BE	0.713	0.736	0.66	0.555	0.600	0.626	0.669	0.602	0.533
		PD	0.457	0.430	0.380	0.303	0.346	0.298	0.376	0.322	0.265
12	-2.4	EX	0.929	0.958	0.911	0.855	0.863	0.915	0.921	0.887	0.849
		ED	0.950	0.955	0.925	0.873	0.893	0.907	0.932	0.900	0.864
	-	NE	0.950	0.970	0.936	0.878	0.894	0.942	0.944	0.923	0.880
		BE	0.889	0.906	0.832	0.751	0.809	0.846	0.841	0.791	0.742
		PD	0.645	0.657	0.591	0.518	0.527	0.513	0.591	0.525	0.450

a Effect size δ.

b EX: Exchangeable correlation, α = (0.03,0.03), ED: Exponential decay correlation, α = (0.03,0.8), NE: Nested exchangeable correlation, α = (0.03,0.015),BE: Block exchangeable correlation, α = (0.03,0.015,0.2),PD: Proportional decay correlation, α = (0.03,0.7).

c Pred: Predicted power, MB: Model-based variance, BC0: uncorrected sandwich estimator of Liang and Zeger (1986), BC1:Bias-corrected sandwich variance of Kauermann and Carroll (2001), BC2: Bias-corrected sandwich variance of Mancl and DeRouen (2001), BC3: Bias-corrected sandwich variance of Fay and Graubard (2001).

D. Simulated results for continuous responses under average intervention effects model

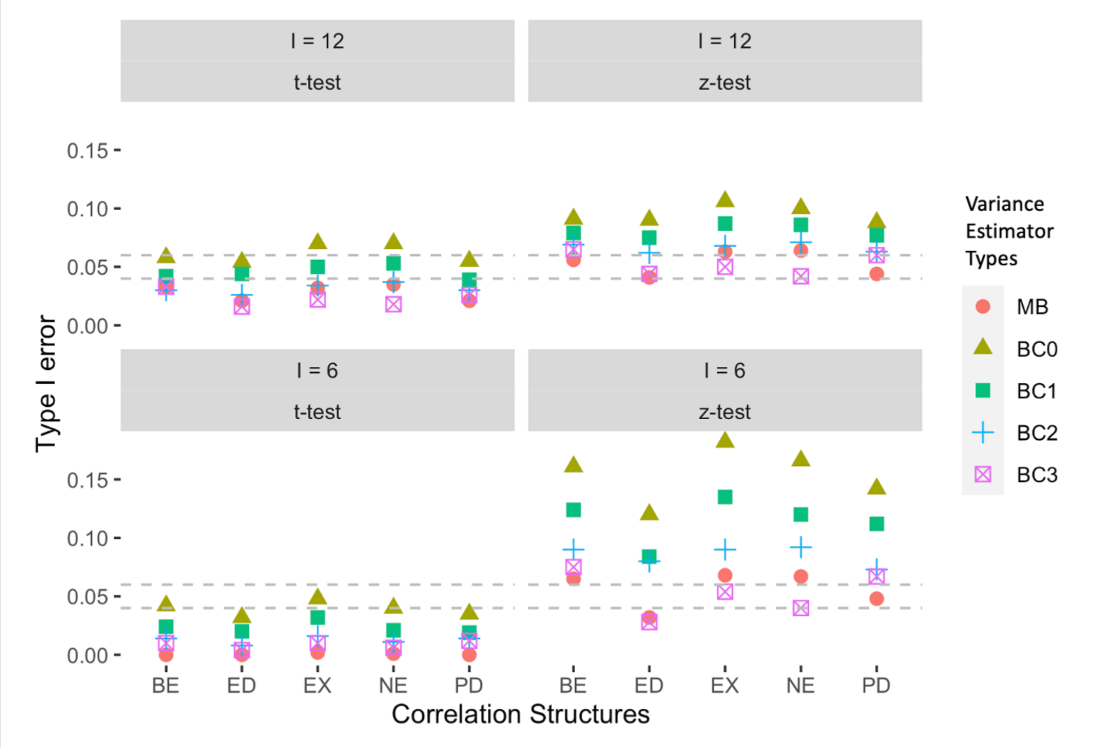

Web Figure 3. Type I error under average intervention effects model of continuous responses. The grey lines indicated an acceptable boundary [4.0%,6.0%] for the empirical test size compared to nominal test size, 0.05. Correlation structures: EX: Exchangeable correlation, ED: Exponential decay correlation, NE: Nested exchangeable correlation,BE: Block exchangeable correlation,PD: Proportional decay correlation. MB: Model-based variance, BC0: uncorrected sandwich estimator of Liang and Zeger (1986), BC1:Bias-corrected sandwich variance of Kauermann and Carroll (2001), BC2: Bias-corrected sandwich variance of Mancl and DeRouen (2001), BC3: Bias-corrected sandwich variance of Fay and Graubard (2001).

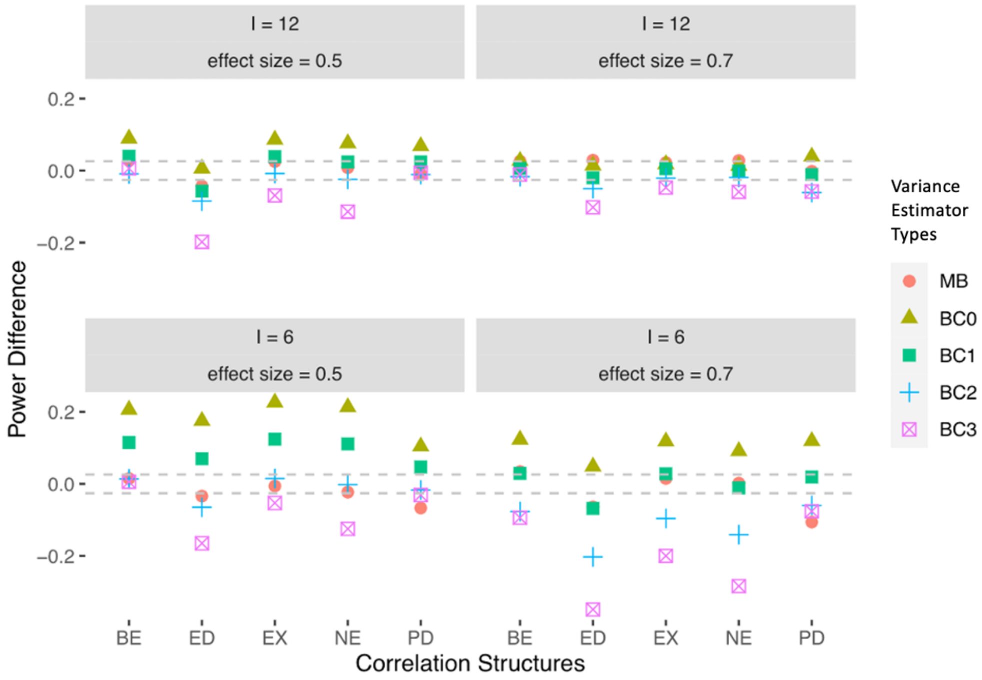

Web Figure 4. Empirical power differences under average intervention effects model using t-test for continuous responses.EX: Exchangeable correlation, ED: Exponential decay correlation,NE: Nested exchangeable correlation,,BE: Block exchangeable correlation,PD: Proportional decay correlation.MB: Model-based variance, BC0: uncorrected sandwich estimator of Liang and Zeger (1986), BC1:Bias-corrected sandwich variance of Kauermann and Carroll (2001), BC2: Bias-corrected sandwich variance of Mancl and DeRouen (2001), BC3: Bias-corrected sandwich variance of Fay and Graubard (2001).

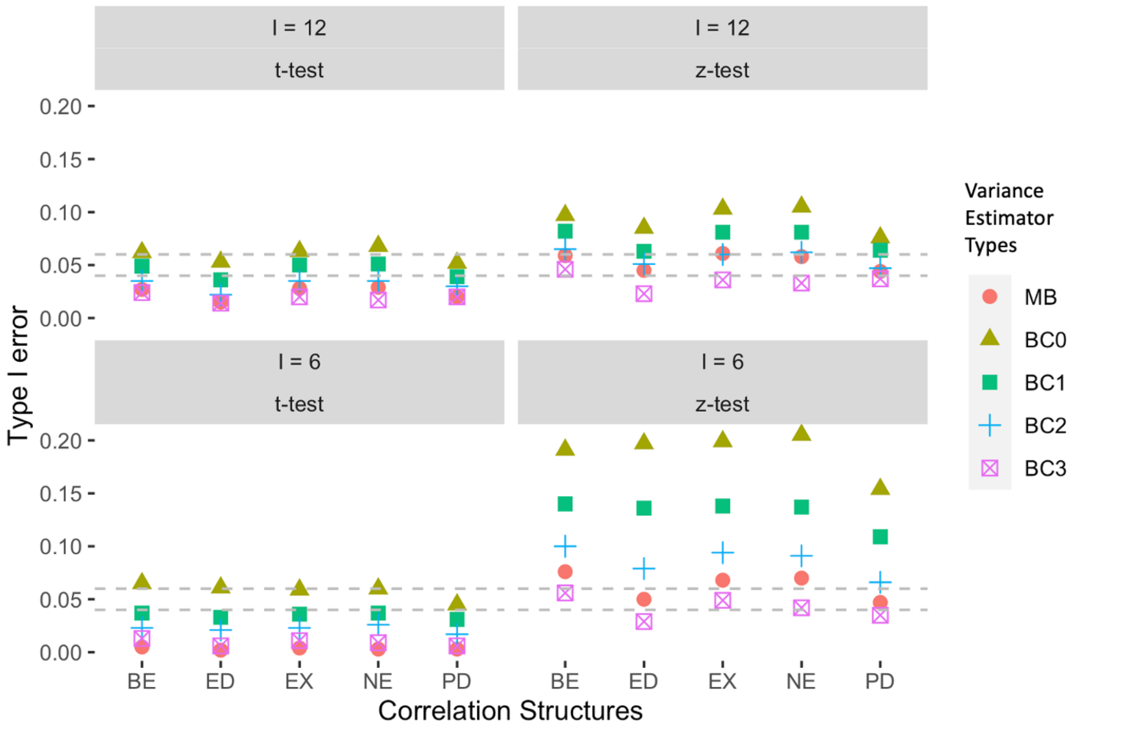

Web Figure 5. Type I error under incremental intervention effects model for continuous responses. The grey lines indicated an acceptable boundary [4.0%,6.0%] for the empirical test size compared to nominal test size, 0.05. Correlation structures: EX: exchangeable, ED: Exponential decay,NE: Nested exchangeable correlation,BE: Block exchangeable correlation,PD: Proportional decay correlation. MB: Model-based variance, BC0: uncorrected sandwich estimator of Liang and Zeger (1986), BC1:Bias-corrected sandwich variance of Kauermann and Carroll (2001), BC2: Bias-corrected sandwich variance of Mancl and DeRouen (2001), BC3: Bias-corrected sandwich variance of Fay and Graubard (2001).

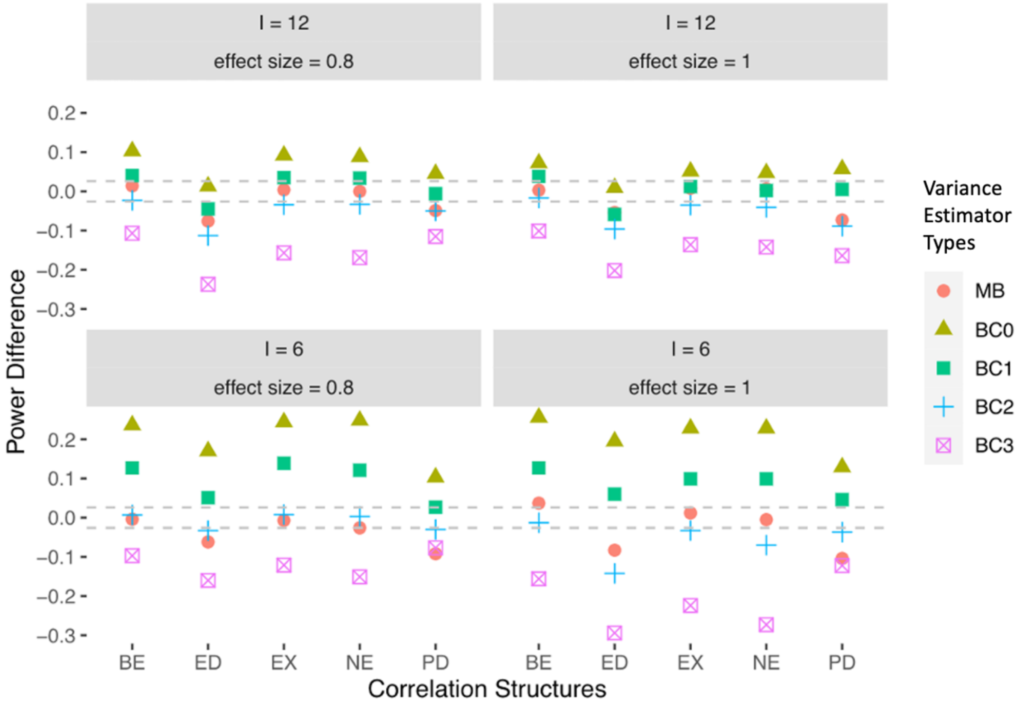

Web Figure 6. Empirical power differences under incremental intervention effects model using t-test for continuous responses.EX: Exchangeable correlation, ED: Exponential decay correlation,NE: Nested exchangeable correlation,BE: Block exchangeable correlation,PD: Proportional decay correlation.MB: Model-based variance, BC0: uncorrected sandwich estimator of Liang and Zeger (1986), BC1:Bias-corrected sandwich variance of Kauermann and Carroll (2001), BC2: Bias-corrected sandwich variance of Mancl and DeRouen (2001), BC3: Bias-corrected sandwich variance of Fay and Graubard (2001).

Web Table 3. Empirical power of GEE analysis using different variance estimators and predicted power under average intervention effects model for continuous responses.

			z-test				t-test				
I	Effect Size[a]	Corr[b]	Pred[c]	MB[c]	BC2[c]	BC3[c]	Pred[c]	MB[c]	BC0[c]	BC1[c]	BC2[c]
6	0.5	EX	0.701	0.728	0.651	0.569	0.268	0.262	0.494	0.392	0.283
		ED	0.714	0.712	0.590	0.428	0.279	0.245	0.454	0.349	0.214
		NE	0.734	0.730	0.664	0.512	0.296	0.273	0.509	0.407	0.294
		BE	0.699	0.716	0.629	0.641	0.267	0.282	0.473	0.382	0.281
		PD	0.485	0.458	0.449	0.416	0.149	0.082	0.253	0.196	0.132
6	0.7	EX	0.936	0.934	0.851	0.808	0.608	0.623	0.726	0.636	0.512
		ED	0.942	0.925	0.801	0.659	0.626	0.562	0.674	0.558	0.423
		NE	0.951	0.936	0.865	0.763	0.653	0.655	0.744	0.642	0.512
		BE	0.935	0.939	0.861	0.878	0.605	0.639	0.728	0.634	0.528
		PD	0.768	0.716	0.667	0.630	0.329	0.223	0.448	0.348	0.269
12	0.5	EX	0.755	0.775	0.736	0.703	0.646	0.671	0.732	0.685	0.638
		ED	0.781	0.738	0.688	0.612	0.676	0.633	0.682	0.619	0.591
		NE	0.787	0.789	0.753	0.683	0.684	0.692	0.760	0.708	0.660
		BE	0.720	0.725	0.717	0.725	0.608	0.636	0.697	0.648	0.599
		PD	0.507	0.484	0.476	0.486	0.392	0.383	0.460	0.416	0.381
12	0.7	EX	0.960	0.971	0.931	0.919	0.909	0.931	0.927	0.914	0.888
		ED	0.969	0.975	0.941	0.911	0.924	0.953	0.938	0.904	0.874
		NE	0.971	0.978	0.943	0.926	0.928	0.956	0.941	0.927	0.909
		BE	0.945	0.954	0.921	0.922	0.887	0.913	0.914	0.893	0.870
		PD	0.791	0.786	0.743	0.745	0.688	0.686	0.727	0.677	0.627

a Effect size δ.

b EX: Exchangeable correlation, α = (0.03,0.03), ED: Exponential decay correlation, α = (0.03,0.8), NE: Nested exchangeable correlation, α = (0.03,0.015),BE: Block exchangeable correlation, α = (0.03,0.015,0.2),PD: Proportional decay correlation, α = (0.03,0.7).

c Pred: Predicted power, MB: Model-based variance, BC0: uncorrected sandwich estimator of Liang and Zeger (1986), BC1:Bias-corrected sandwich variance of Kauermann and Carroll (2001), BC2: Bias-corrected sandwich variance of Mancl and DeRouen (2001), BC3: Bias-corrected sandwich variance of Fay and Graubard (2001).

Web Table 4. Empirical power of GEE analysis using different variance estimators and predicted power under incremental intervention effects model for continuous responses.

			z-test				t-test				
**I**	**Effect Size**[a]	**Corr**[b]	**Pred**[c]	**MB**[c]	**BC2**[c]	**BC3**[c]	**Pred**[c]	**MB**[c]	**BC0**[c]	**BC1**[c]	**BC2**[c]
6	0.8	EX	0.661	0.667	0.595	0.406	0.239	0.232	0.483	0.378	0.247
		ED	0.697	0.620	0.557	0.321	0.265	0.203	0.435	0.316	0.232
		NE	0.707	0.700	0.617	0.404	0.273	0.247	0.521	0.394	0.276
		BE	0.641	0.652	0.588	0.439	0.226	0.222	0.462	0.353	0.233
		PD	0.405	0.332	0.284	0.185	0.120	0.028	0.223	0.147	0.090
6	1.0	EX	0.843	0.842	0.742	0.581	0.422	0.434	0.650	0.521	0.389
		ED	0.871	0.827	0.755	0.464	0.467	0.384	0.662	0.527	0.325
		NE	0.879	0.879	0.788	0.576	0.481	0.476	0.709	0.580	0.411
		BE	0.827	0.850	0.769	0.603	0.399	0.436	0.655	0.526	0.386
		PD	0.575	0.488	0.408	0.263	0.189	0.085	0.318	0.235	0.152
12	0.8	EX	0.727	0.726	0.695	0.592	0.616	0.620	0.708	0.651	0.582
		ED	0.770	0.705	0.672	0.561	0.664	0.588	0.677	0.619	0.551
		NE	0.770	0.771	0.733	0.623	0.664	0.664	0.752	0.698	0.631
		BE	0.665	0.665	0.650	0.567	0.548	0.562	0.651	0.588	0.525
		PD	0.424	0.366	0.352	0.294	0.317	0.268	0.362	0.311	0.267
12	1.0	EX	0.894	0.893	0.851	0.791	0.815	0.823	0.866	0.827	0.780
		ED	0.921	0.889	0.836	0.751	0.852	0.798	0.861	0.793	0.756
		NE	0.921	0.920	0.880	0.809	0.853	0.859	0.900	0.855	0.812
		BE	0.847	0.851	0.827	0.779	0.755	0.758	0.827	0.793	0.738
		PD	0.599	0.535	0.535	0.441	0.480	0.407	0.537	0.485	0.391

a Effect size δ.

b EX: Exchangeable correlation, α = (0.03,0.03), ED: Exponential decay correlation, α = (0.03,0.8), NE: Nested exchangeable correlation, α = (0.03,0.015),BE: Block exchangeable correlation, α = (0.03,0.015,0.2),PD: Proportional decay correlation, α = (0.03,0.7).

c Pred: Predicted power, MB: Model-based variance, BC0: uncorrected sandwich estimator of Liang and Zeger (1986), BC1:Bias-corrected sandwich variance of Kauermann and Carroll (2001), BC2: Bias-corrected sandwich variance of Mancl and DeRouen (2001), BC3: Bias-corrected sandwich variance of Fay and Graubard (2001).

E.Convergence rates for simulated results

Web Table 5. Convergence rate (percentage of convergence out of 2000 replicates) for empirical type I error

**Type**[a]	**I**[b]	**Model**[c]	EX[d]	ED[d]	NE[d]	BE[d]	PD[d]
Binary	6	Average	80.90	32.35	67.75	91.05	56.75
		Incremental	78.05	29.85	64.95	89.85	56.75
	12	Average	99.90	45.30	99.65	99.90	56.60
		Incremental	99.80	44.20	99.55	99.85	57.15
Continuous	6	Average	83.70	24.85	71.80	92.40	57.30
		Incremental	83.30	23.90	72.85	92.70	57.20
	12	Average	99.80	38.70	99.80	100.00	56.75
		Incremental	99.80	38.95	99.70	100.00	56.25

a Responses type: binary responses or continuous responses.

b Number of clusters: 6 clusters or 12 clusters.

c Marginal models: Average: average intervention effects model, Incremental: incremental intervention effects model.

d Correlation structures: EX: Exchangeable correlation, α = (0.03,0.03), ED: Exponential decay, α = (0.03,0.8), NE: Nested exchangeable correlation, α = (0.03,0.015), BE: Block exchangeable correlation, α = (0.03,0.015,0.2),PD: Proportional decay correlation, α = (0.03,0.7).

Web Table 6. Convergence rate percentage of convergence out of 1000 replicates) for empirical power

**Type**[a]	**I**[b]	**Model**[c]	**Effect size**[d]	EX[e]	ED[e]	NE[e]	BE[e]	PD[e]
Binary	6	Average	-1.2	82.2	32.9	68.4	89.0	54.0
			-1.4	82.3	31.4	69.1	87.8	54.5
		Incremental	-1.8	76.5	29.5	65.0	86.8	55.1
			-2.4	76.6	30.9	64.3	80.0	53.2
	12	Average	-1.2	100.0	47.5	99.5	100.0	57.1
			-1.4	100.0	46.9	99.7	100.0	54.1
		Incremental	-1.8	100.0	46.6	99.9	99.7	57.7
			-2.4	99.8	44.2	97.9	98.9	57.5
Continuous	6	Average	0.5	80.8	22.9	70.3	92.1	57.0
			0.7	84.4	26.7	74.6	93.4	59.2
		Incremental	0.8	83.9	23.7	72.1	94.0	57.8
			1.0	83.7	23.7	73.1	94.5	57.8
	12	Average	0.5	100	38.1	99.7	100	58.0
			0.7	100	40.5	99.9	100	57.6
		Incremental	0.8	100	39.6	100	100	58.2
			1.0	100	39.7	100	100	58.5

a Responses type: binary responses or continuous responses.

b Number of clusters: 6 clusters or 12 clusters.

c Marginal models: Average: average intervention effects model, Incremental: incremental intervention effects model.

d Intervention effect size of marginal model.

e Correlation structures: EX: Exchangeable correlation, α = (0.03,0.03), ED: Exponential decay, α = (0.03,0.8), NE: Nested exchangeable correlation, α = (0.03,0.015), BE: Block exchangeable correlation, α = (0.03,0.015,0.2),PD: Proportional decay correlation, α = (0.03,0.7).

F. The application of fast GEE method for continuous responses to six SW-CRTs

SNF	Period = 1 month																				
	1	2	3	4	5	6	7	8	9	10	11	12	13	14	15	16	17	18	19	20	21	22
1	4	4	4	4	4			4	4	4	4	4	4	4	4	4	4					
2		4	4	4	4	4	4			4	4	4	4	4	4	4	4	4				
3			4	4	4	4	4	4	4			4	4	4	4	4	4	4	4			
4				4	4	4	4	4	4	4	4			4	4	4	4	4	4	4		
5					4	4	4	4	4	4	4	4	4			4	4	4	4	4	4	
6						4	4	4	4	4	4	4	4	4	4			4	4	4	4	4

Web Figure 7. Design Pattern of Design A, the Connect-Home study

SNF	Period = 1 month																				
	1	2	3	4	5	6	7	8	9	10	11	12	13	14	15	16	17	18	19	20	21	22
1	4	4	4	4	4		4	4	4	4	4	4	4	4	4	4						
2		4	4	4	4	4	4		4	4	4	4	4	4	4	4	4					
3			4	4	4	4	4	4	4		4	4	4	4	4	4	4	4				
4				4	4	4	4	4	4	4	4		4	4	4	4	4	4	4			
5					4	4	4	4	4	4	4	4	4		4	4	4	4	4	4		
6						4	4	4	4	4	4	4	4	4	4		4	4	4	4	4	

Web Figure 8. Design Pattern of Design B, an incomplete SW-CRT with one implementation period, staggered entry and drop out

SNF	Period = 1 month																				
	1	2	3	4	5	6	7	8	9	10	11	12	13	14	15	16	17	18	19	20	21	22
1		4	4	4	4	4	4	4	4	4	4	4	4	4	4	4						
2			4	4	4	4	4	4	4	4	4	4	4	4	4	4	4					
3				4	4	4	4	4	4	4	4	4	4	4	4	4	4	4				
4					4	4	4	4	4	4	4	4	4	4	4	4	4	4	4			
5						4	4	4	4	4	4	4	4	4	4	4	4	4	4	4		
6							4	4	4	4	4	4	4	4	4	4	4	4	4	4	4	

Web Figure 9. Design Pattern of Design C, an incomplete SW-CRT only with staggered entry and drop out

SNF	Period = 1 month																				
	1	2	3	4	5	6	7	8	9	10	11	12	13	14	15	16	17	18	19	20	21	22
1			2	4	4			4	4	4	4	4	4	4	4	4	4	4	4	2		
2			2	4	4	4	4			4	4	4	4	4	4	4	4	4	4	2		
3			2	4	4	4	4	4	4			4	4	4	4	4	4	4	4	2		
4			2	4	4	4	4	4	4	4	4			4	4	4	4	4	4	2		
5			2	4	4	4	4	4	4	4	4	4	4			4	4	4	4	2		
6			2	4	4	4	4	4	4	4	4	4	4	4	4			4	4	2		

Web Figure 10. Design Pattern of Design D, an incomplete SW-CRT with two implementation periods

SNF	Period = 1 month																				
	1	2	3	4	5	6	7	8	9	10	11	12	13	14	15	16	17	18	19	20	21	22
1			2	4	4		4	4	4	4	4	4	4	4	4	4	4	4	2			
2			2	4	4	4	4		4	4	4	4	4	4	4	4	4	4	2			
3			2	4	4	4	4	4	4		4	4	4	4	4	4	4	4	2			
4			2	4	4	4	4	4	4	4	4		4	4	4	4	4	4	2			
5			2	4	4	4	4	4	4	4	4	4	4		4	4	4	4	2			
6			2	4	4	4	4	4	4	4	4	4	4	4	4		4	4	2			

Web Figure 11. Design Pattern of Design E, an incomplete SW-CRT with one implementation period

SNF	Period = 1 month																				
	1	2	3	4	5	6	7	8	9	10	11	12	13	14	15	16	17	18	19	20	21	22
1				2	4	4	4	4	4	4	4	4	4	4	4	4	4	4	2			
2				2	4	4	4	4	4	4	4	4	4	4	4	4	4	4	2			
3				2	4	4	4	4	4	4	4	4	4	4	4	4	4	4	2			
4				2	4	4	4	4	4	4	4	4	4	4	4	4	4	4	2			
5				2	4	4	4	4	4	4	4	4	4	4	4	4	4	4	2			
6				2	4	4	4	4	4	4	4	4	4	4	4	4	4	4	2			

Web Figure 12. Design Pattern of Design F, a complete SW-CRT

Web Table 7. The predicted GEE power of the t-test for continuous responses in cross-sectional SW-CRTs having six sequences, 16 to 22 periods, and a total of 360 participants with a maximum of four participants per cluster-period

Model[b]	I	Corr[c]	A[a]	B[a]	C[a]	D[a]	E[a]	F[a]
Staggered Entry/Termination			Yes	Yes	Yes	No	No	No
Number of Implementation periods			2	1	0	2	1	0
Average	6	EX	0.268	0.331	0.412	0.297	0.361	0.440
		ED	0.279	0.342	0.424	0.273	0.337	0.421
		NE	0.296	0.363	0.446	0.309	0.377	0.458
	12	EX	0.646	0.720	0.791	0.663	0.734	0.801
		ED	0.676	0.745	0.812	0.665	0.738	0.808
		NE	0.684	0.754	0.819	0.686	0.756	0.821
Incremental	6	EX	0.422	0.447	0.483	0.620	0.637	0.665
		ED	0.467	0.496	0.532	0.627	0.650	0.680
		NE	0.481	0.515	0.559	0.668	0.691	0.723
	12	EX	0.815	0.836	0.862	0.914	0.924	0.936
		ED	0.852	0.871	0.892	0.928	0.938	0.949
		NE	0.853	0.873	0.896	0.933	0.943	0.954

a A-F: Designs A to F are shown in Appendix F for six clusters. For twelve clusters, the cluster-period sizes are half the amount shown, i.e., 1 and 2 instead of 2 and 4.

b Marginal Models: Average: average intervention effects model with $\delta = 0.5$, Incremental: incremental intervention effects model with $\delta = 1$ and maximum intervention periods equaling to 10.

c Correlation structures: EX: Exchangeable correlation, α = (0.03,0.03), ED: Exponential decay, α = (0.03,0.8), NE: Nested exchangeable correlation, α = (0.03,0.015)

Web Table 8. Fast GEE power using t-test under different cluster period size for Connect-Home trial with 6 clusters and continuous responses.

			Cluster-period size[d]					
**Model**[a]	**Effect Size**[b]	**Corr**[c]	4	5	6	7	8	9
Average	0.5	EX	0.268	0.339	0.411	0.482	0.548	0.608
		ED	0.279	0.341	0.399	0.451	0.498	0.540
		NE	0.296	0.369	0.440	0.504	0.563	0.614
Average	0.7	EX	0.608	0.714	0.790	0.842	0.879	0.906
		ED	0.626	0.716	0.778	0.821	0.852	0.875
		NE	0.653	0.749	0.813	0.856	0.886	0.908
Incremental	0.8	EX	0.239	0.297	0.356	0.416	0.474	0.530
		ED	0.265	0.321	0.372	0.418	0.459	0.495
		NE	0.273	0.337	0.399	0.457	0.510	0.558
Incremental	1.0	EX	0.422	0.519	0.605	0.676	0.735	0.782
		ED	0.467	0.555	0.624	0.678	0.720	0.753
		NE	0.481	0.579	0.657	0.719	0.766	0.803

a Marginal Models: Average: average intervention effects model, Incremental: incremental intervention effects model.

b Effect Size: δ.

c Correlation structures: EX: Exchangeable correlation, α = (0.03,0.03), ED: Exponential decay, α = (0.03,0.8), NE: Nested exchangeable correlation, α = (0.03,0.015).

d Cluster-period size: the number of participants in a cluster-period.

G. The SAS macro: CRTFASTGEEPWR

G.1: Details of the input options and outputs variables in the SAS macro

Web Table 9. Option arguments in the SAS macro CRTFASTGEEPWR

Macro Variable	Input	Description
ALPHA	Variable	Significance level, default at 0.05 (optional)
M	Variable	Number of clusters in each sequence, a vector with dimension S*T
CP_SIZE_MATRIX	Variable	Number of cluster-period sizes, a matrix vector with dimension S*T
INTERVENTION_EFFECT_TYPE	=AVE	Average intervention effects model
	=INC	Incremental intervention effects model
PERIOD_EFFECT_TYPE	=CAT	Categorical time period model
	=LIN	Linear period effects model
DELTA	Variable	The parameters of intervention
BETA_PERIOD_EFFECTS	Variable	The parameters for period effects:<br>A T*1 vector for categorical period effect<br>A 2*1 vector for continuous period effect
CORR_TYPE	=NE	Nested exchangeable
	=ED	Exponential decay
	=BE	Block exchangeable
	=PD	Proportional decay
ALPHA0	Variable	The within-period correlation in exponential decay and proportional decay correlation structure
R0	Variable	Correlation decay rate over time in exponential decay and proportional decay correlation structure
ALPHA1	Variable	The within-period correlation in nested exchangeable and block exchangeable correlation structure
ALPHA2	Variable	The inter-period correlation in nested exchangeable and block exchangeable
ALPHA3	Variable	The within-subject correlation in block exchangeable correlation structure
MAX_INTERVENTION_PERIOD	Variable	The number of maximum intervention periods in incremental intervention effect model (optional)
DIST	=binary<br>=normal<br>=poisson	The distribution for the outcomes
LINK	=log<br>=logit<br>=identity	The link function for the outcomes,<br>canonical link as the default choice<br>(optional)
PHI	Variable	The dispersion parameter
DESIGNPATTERN	Variable	A matrix with dimension S x T of 0, 1 and 2,<br>with 0 representing control periods,<br>1 standing for intervention periods,<br>2 presenting periods without data collection.
DF_CHOICE	=1	degree of freedom(df), df = I-p, p is the number of parameters in marginal model
	=2	df = I-2 (optional)

Web Table 10. Output information in the SAS macro CRTFASTGEEPWR

Output Variable	Descriptions
T	Number of periods, a scalar
S	Number of sequences (steps), a scalar
clusters	Number of clusters in the CRT
df	Degree of freedom in the test of the intervention effects
theta	Estimated number of parameters in marginal model
totaln	Total number of the participants in the CRT
Dist	The distribution for the outcomes
link	The link function used in the GEE model
stddel	Estimated standard deviation of the intervention effects
zpower	Power using the z-test
tpower	Power using the t-test

G.2: Examples in using the SAS macro CRTFASTGEEPWR

The first example is to replicate the power calculation in the application section in Table 3 for the Connect-Home study (Design A) with binary outcomes and 6 clusters, under average intervention effects model and nested exchangeable correlation structure. The details of the parameter specifications are in the following code.

```
%CRTFASTGEEPWR(alpha=0.05, m =%str(J(6,1,1)), corr_type = NE,
alpha1 = 0.03, alpha2 = 0.015 ,intervention_effect_type=AVE,
delta = -1.2, period_effect_type=LIN,
beta_period_effects =%str({0.85,-0.01}) ,dist = binary,phi=1,
   CP_size_matrix =
  %str({4 4 4 4 4 0 0 4 4 4 4 4 4 4 4 4 4 0 0 0 0 0,
        0 4 4 4 4 4 4 0 0 4 4 4 4 4 4 4 4 4 0 0 0 0,
        0 0 4 4 4 4 4 4 4 0 0 4 4 4 4 4 4 4 4 0 0 0,
        0 0 0 4 4 4 4 4 4 4 4 0 0 4 4 4 4 4 4 4 0 0,
        0 0 0 0 4 4 4 4 4 4 4 4 4 0 0 4 4 4 4 4 4 0,
        0 0 0 0 0 4 4 4 4 4 4 4 4 4 4 0 0 4 4 4 4 4}),
   DesignPattern =
 %str({0 0 0 0 0 2 2 1 1 1 1 1 1 1 1 1 1 2 2 2 2 2,
        2 0 0 0 0 0 0 2 2 1 1 1 1 1 1 1 1 1 2 2 2 2,
        2 2 0 0 0 0 0 0 0 2 2 1 1 1 1 1 1 1 1 2 2 2,
        2 2 2 0 0 0 0 0 0 0 0 2 2 1 1 1 1 1 1 1 2 2,
        2 2 2 2 0 0 0 0 0 0 0 0 0 2 2 1 1 1 1 1 1 2,
        2 2 2 2 2 0 0 0 0 0 0 0 0 0 0 2 2 1 1 1 1 1}));
```

Figure 13: Output of the Example 1

```
The fast GEE power of binary outcomes with nested exchangeable
correlation structure and (alpha1,alpha2):(0.03, 0.015)
Under average intervention effects model and delta = -1.2

 T  S clusters df theta  totaln Dist  Link     stddel    zpower    tpower

22  6    6     3  0.85    360  binary logit  2.9589846 0.8411076  0.418764
                 -0.01
                 -1.2
```

The second example is to replicate the power calculation in the application section in Web Table 7 for the design F (complete SW-CRT) with normal outcomes and 12 clusters, under incremental intervention effects model and exponential decay correlation structure. The details of the parameter specifications are in the following code.

```
%CRTFASTGEEPWR(alpha=0.05, m =%str(J(6,1,2)), corr_type = ED,
alpha0 = 0.03,r0 = 0.8 ,intervention_effect_type=INC, delta =1,
max_intervention_period=10, period_effect_type=LIN,
beta_period_effects =%str({2,-0.05}),
dist =normal,phi=1,

    CP_size_matrix =
  %str({1 2 2 2 2 2 2 2 2 2 2 2 2 2 2 1,
        1 2 2 2 2 2 2 2 2 2 2 2 2 2 2 1,
        1 2 2 2 2 2 2 2 2 2 2 2 2 2 2 1,
        1 2 2 2 2 2 2 2 2 2 2 2 2 2 2 1,
        1 2 2 2 2 2 2 2 2 2 2 2 2 2 2 1,
        1 2 2 2 2 2 2 2 2 2 2 2 2 2 2 1}),

   DesignPattern =
  %str({0 0 0 1 1 1 1 1 1 1 1 1 1 1 1 1,
        0 0 0 0 0 1 1 1 1 1 1 1 1 1 1 1,
        0 0 0 0 0 0 0 1 1 1 1 1 1 1 1 1,
        0 0 0 0 0 0 0 0 0 1 1 1 1 1 1 1,
        0 0 0 0 0 0 0 0 0 0 0 1 1 1 1 1,
        0 0 0 0 0 0 0 0 0 0 0 0 0 1 1 1}));
```

Figure 14: Output of the Example 2

```
The fast GEE power of normal outcomes with exponential decay
correlation structure and (alpha0,r0):(0.03, 0.8)
Under incremental intervention effects model and delta = 1

 T  S clusters df theta  totaln Dist   Link     stddel    zpower    tpower
16  6   12     9  2        360  normal identity 4.0810388 0.9830422 0.9488577
                  -0.05
                  1
```

The third example is to replicate the power calculation for complete SW-CRT with categorical period effects. There are 4 steps and five periods in the SW-CRT, with 24 clusters and 12000 subjects in total. The categorical periods effects are assumed to be constant overtime and the the power is calculated under average intervention effects model and exchangeable correlation structure. Details of the parameter specifications are in the attached SAS code.

```
%CRTFASTGEEPWR(alpha=0.05, m =%str(J(4,1,6)), corr_type = NE,
   intervention_effect_type=AVE, delta =-0.465057,
   period_effect_type=CAT, beta_period_effects =%str(J(5,1,-2.944439)),
   alpha1 = 0.01,alpha2 = 0.01, dist = binary,phi=1,
   CP_size_matrix =  %str(J(4,5,100)),
   DesignPattern =
   %str({0 1 1 1 1,
         0 0 1 1 1,
         0 0 0 1 1,
         0 0 0 0 1}));
```

Figure 15: Output of the Example 3

```
The fast GEE power of binary outcomes with nested exchangeable
correlation structure and (alpha1,alpha2):(0.01, 0.01)
Under average intervention effects model and delta = -0.465057

T   S clusters df theta     totaln Dist   Link     stddel    zpower    tpower
5   4   24     18 -2.944439 12000 binary logit 2.8489842 0.8130039 0.7679575
                  -2.944439
                  -2.944439
                  -2.944439
                  -2.944439
                  -0.465057
```

H: Literature on SW-CRTs with GEE-type marginal model analysis of SW-CRTs with small to moderate number of clusters

Web Table 11. Simulation settings and recommended bias-corrected variance estimators for GEE analysis of SW-CRTs

Papers	Design types	Incomplete Design	Outcome types	Number of clusters (I)	True Corr^a	Working Corr^a	Objectives	Recommended BC^b
Individual level analysis								
Ford and Westgate[1]	Cross-sectional	No	Binary	3-24	EX, BE	EX	Type I error	AVG(BC1,BC2), df = $I-2$
Thompson[2]	Cross-sectional	No	Binary	6-54	EX, AR(1)	IND, EX	Power, Bias of intervention effect estimation	BC1, BC3 df $= I-p, I \geq 18$ df$^c_{FG}$, $I \geq 12$
Li et al.[3]	Cohort	No	Continuous, binary	8-25	BE	BE	Type I error and power	BC1 or BC3, df $= I-p$
Li[4]	Cohort	No	Continuous	9-24	PD	PD	Type I error, power	BC1, df $= I-2$
Zhang et al.[5]	Cohort, cross-sectional	Yes	Continuous, binary	6,12	EX,NE,BE, ED,PD	EX,NE,BE, ED,PD	Type I error, power	BC1, df = $I-p$
Cluster-period level analysis								
Scott et al.[6]	Cohort	No	Continuous	10,20,50	AR(1)	AR(1), EX	Type I error, power	BC3, df$^c_{FG}$
Li, Yu et al.[7]	Cohort	No	Binary	12, 24, 36	NE,ED	NE,ED	Bias of intervention effect, ICC estimations	BC1, df $= I-2$

a: IND: Independent correlation structure, EX: Exchangeable correlation, ED: Exponential decay correlation, NE: Nested exchangeable correlation, BE: Block exchangeable correlation,PD: Proportional decay correlation.

b: Recommend Bias-Corrected variance estimators (BC) for type I error or controlling bias of intervention effect estimation by the results of simulation studies; BC1:Bias-corrected sandwich variance of Kauermann and Carroll (2001), BC2: Bias-corrected sandwich variance of Mancl and DeRouen (2001), BC3: Bias-corrected sandwich variance of Fay and Graubard (2001).

c: df$_{FG}$ is from the degree freedom of Fay and Graubard (2001) and it uses the weighted average of covariance estimates of the terms of the estimating equation to calculate the degree of freedom.

References

1. Ford WP and Westgate PM. Maintaining the validity of inference in small-sample stepped wedge cluster randomized trials with binary outcomes when using generalized estimating equations. *Statistics in Medicine* 2020; 39(21): 2779–2792. https://doi.org/10.1002/sim.8575. https://onlinelibrary.wiley.com/doi/abs/10.1002/sim.8575. https://onlinelibrary.wiley.com/doi/pdf/10.1002/sim.8575.
2. Thompson JA, Hemming K, Forbes A et al. Comparison of small-sample standard-error corrections for generalised estimating equations in stepped wedge cluster randomised trials with a binary outcome: A simulation

study. *Statistical methods in medical research* 2021; 30: 425–439.

3. Li F, Turner EL and Preisser JS. Sample size determination for gee analyses of stepped wedge cluster randomized trials. *Biometrics* 2018; 74(4): 1450–1458.
4. Li F. Design and analysis considerations for cohort stepped wedge cluster randomized trials with a decay correlation structure. *Statistics in medicine* 2020; 39(4): 438–455.
5. Zhang Y, Preisser JS, Turner EL, Rathouz PJ, Toles M, Li F. A general method for calculating power for GEE analysis of complete and incomplete stepped wedge cluster randomized trials. Submitted to Statistical Methods in Medical Research.
6. Scott JM, deCamp A, Juraska M et al. Finite-sample corrected generalized estimating equation of population average treatment effects in stepped wedge cluster randomized trials. *Statistical methods in medical research* 2017; 26: 583–597.
7. Li, F, Yu, H, Rathouz, P, Turner, E & Preisser, JS Marginal modeling of cluster-period means and intraclass correlations in stepped wedge designs with binary outcomes. *Biostatistics* 2021; 23(3): 772–788.
